# miRWoods: Enhanced precursor detection and stacked random forests for the sensitive detection of microRNAs

**DOI:** 10.1371/journal.pcbi.1007309

**Published:** 2019-10-09

**Authors:** Jimmy Bell, Maureen Larson, Michelle Kutzler, Massimo Bionaz, Christiane V. Löhr, David Hendrix

**Affiliations:** 1 School of Electrical Engineering and Computer Science, Oregon State University, Corvallis, OR, United States of America; 2 Departments of Clinical and Biomedical Sciences, College of Veterinary Medicine, Oregon State University, Corvallis, OR, United States of America; 3 Department of Animal and Rangeland Sciences, Oregon State University, Corvallis, OR, United States of America; 4 Department of Biochemistry and Biophysics, Oregon State University, Corvallis, OR, United States of America; University of California Irvine, UNITED STATES

## Abstract

MicroRNAs are conserved, endogenous small RNAs with critical post-transcriptional regulatory functions throughout eukaryota, including prominent roles in development and disease. Despite much effort, microRNA annotations still contain errors and are incomplete due especially to challenges related to identifying valid miRs that have small numbers of reads, to properly locating hairpin precursors and to balancing precision and recall. Here, we present miRWoods, which solves these challenges using a duplex-focused precursor detection method and stacked random forests with specialized layers to detect mature and precursor microRNAs, and has been tuned to optimize the harmonic mean of precision and recall. We trained and tuned our discovery pipeline on data sets from the well-annotated human genome, and evaluated its performance on data from mouse. Compared to existing approaches, miRWoods better identifies precursor spans, and can balance sensitivity and specificity for an overall greater prediction accuracy, recalling an average of 10% more annotated microRNAs, and correctly predicts substantially more microRNAs with only one read. We apply this method to the under-annotated genomes of *Felis catus* (domestic cat) and *Bos taurus* (cow). We identified hundreds of novel microRNAs in small RNA sequencing data sets from muscle and skin from cat, from 10 tissues from cow and also from human and mouse cells. Our novel predictions include a microRNA in an intron of tyrosine kinase 2 (TYK2) that is present in both cat and cow, as well as a family of mirtrons with two instances in the human genome. Our predictions support a more expanded miR-2284 family in the bovine genome, a larger mir-548 family in the human genome, and a larger let-7 family in the feline genome.

## Author summary

While the computational prediction of microRNA loci from high-throughput sequence data is well-studied, challenges persist in defining the minimum number of reads required for a locus to be evaluated, as well as in defining the precursor span. We present a new method, “miRWoods”, which has greater recall of known microRNAs, while also achieving as good or better overall performance. Our approach uses improved duplex-based methods of precursor detection and a pair of random forest layers that sensitively detect mature products and precursors. We trained our model on data from human, and confirmed that it can successfully be applied cross-species by evaluating predictions for the mouse genome. We then applied our approach to new sequencing data mapped to the under-annotated genomes of cow and cat. We were able to use miRWoods to improve annotations for cat and cow microRNAs, and found novel microRNAs in human and mouse, and identified errors in current annotations.

## Introduction

MicroRNAs (miRNAs, miRs) are a highly-conserved class of small endogenous RNA molecules that are involved in post-transcriptional gene silencing by acting as a guide RNA for the RNA-induced silencing complex (RISC). The biogenesis of microRNAs begins with the generation of a primary transcript (pre-miR), which folds into a structure containing one or more ~70-nt hairpins. These hairpin precursors (pre-miRs) are cut at the base by Drosha [1]. After export from the nucleus, the loop of the hairpin is cut by Dicer. The resultant double-stranded RNA duplex is unwound to produce two mature ~22-nt microRNAs (miRs), named 5′ and 3′ after the arm of the hairpin from which they derive. Typically, only one of the mature microRNAs is incorporated into RISC, and the other microRNA is degraded and designated miR-star or miR*. The seed sequence at positions 2–8 of RISC-bound mature microRNAs binds to complementary sequences in the 3′ untranslated regions (UTRs) of mRNAs.

Initially discovered through genetic screens in *C*. *elegans* [2], the advent of deep sequencing data has enabled the high-throughput discovery and annotation of novel microRNAs. Most microRNA prediction approaches begin by aligning size-selected deep sequenced RNA (small RNA-seq) reads to the genome, and then the identification of overlapping aligned reads, “read stacks”. These read stacks correspond to mature microRNA products, as well as other sequenced fragments including microRNA offset RNAs (moRs) [3], hairpin loops, and spurious RNA fragments. The RNA secondary structures for the genomic sequences surrounding the read stacks are predicted and reads overlapping predicted hairpin structures are analyzed for arrangements consistent with microRNA processing. The prediction methods vary in the specifics of how the data are processed, and relevant features are quantified, as well as what classification techniques are used. Methods employing this strategy include miRTRAP [4], the software upon which miRWoods was built, along with miRDeep [5], the improved miRDeep2 [6] and other variants [7, 8], miReap [9], and miRAnalyzer [10].

Several challenges remain in the computational prediction of microRNAs. Current approaches have strengths and weaknesses; while some approaches focus on higher precision at the expense of false negatives, others focus on higher recall at the expense of false positives. Most approaches require a minimum number of mapped reads at a given locus, meaning that many valid lowly expressed microRNAs are missed. Also, hairpin precursor detection is challenging because slight changes in the boundaries can shift the secondary structure prediction away from the hairpin. Our analysis of the predictions from available methods identifies many cases that partially overlap with or are shifted from annotated loci, and mistake 5′ for 3′ mature miRs.

**Fig 1:**
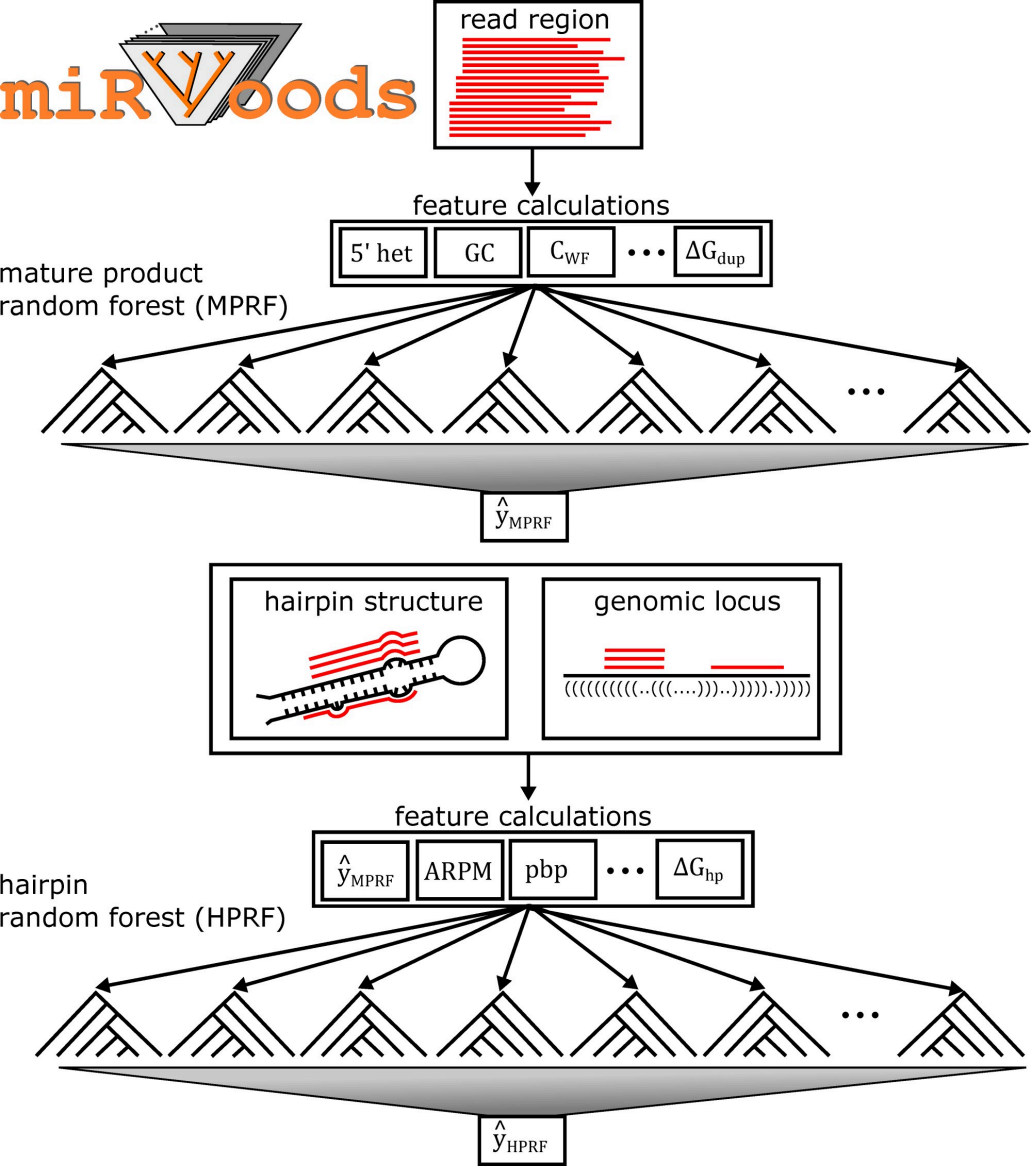
Outline of miRWoods Pipeline. After aligning to the genome, overlapping reads are grouped together to form read stacks. Read stacks are scored used Mature Product Random Forest (MPRF), to predict a set of putative mature microRNAs. Products which meet the minimum threshold score for the MPRF are combined with the surrounding region to form hairpins and each hairpin is folded. Hairpins are scored using the Hairpin Random Forest (HPRF) and a set of final predictions are generated which meet the minimum threshold for the HPRF score.

These remaining challenges to microRNA discovery motivated us to create miRWoods, a microRNA discovery pipeline using stacked random forests with an improved method for determining hairpin precursor span (Fig 1). The miRWoods pipeline consists of a mature product random forest (MPRF) for mature product detection, and a hairpin precursor random forest (HPRF) for hairpin precursor identification. For, balancing precision *versus* recall, we tuned miRWoods to optimize F-score, which is the harmonic mean of precision and recall. We trained and tuned miRWoods on well-annotated human data sets, evaluated cross-species performance using mouse data and used the pipeline to subsequently identify novel microRNAs in the feline and bovine genomes.

## Results

### Overview of strategy behind miRWoods

Because current approaches impose a threshold for the read abundance for a locus to be evaluated as a putative microRNA, many low-abundant miRs are missed. To avoid this, we have added a machine learning classifier to identify read stacks that are plausible mature microRNA loci, thereby enabling miRWoods to detect microRNAs with a single read. This RF evaluates read abundance-related features in the context of other features to classify plausible mature products (Table 1). To avoid the sensitive-dependence on precursor span for secondary structure prediction, we examine several putative precursors for each read stack, including one derived from the boundaries of the optimal duplex between the read stack and surrounding genomic region (duplex-focused spans) and those derived from the boundaries with other products (product-focused spans). Through extensive feature-engineering, we have added several novel features to help classify the microRNA precursors, which are listed in Table 2. Finally, we have tuned parameters of our model to optimize F-score, the harmonic mean of precision and recall, to result in improved performance that doesn’t sacrifice precision, and recalls 10% more annotated microRNAs on average.

**Table 1:** Features used in the mature products random forest (MRPF).

fivePrimeHet	5′-heterogeneity of product reads
medianLength	Median length of product reads
gcContent	GC content of product sequence
aa,ac,ag,at,ca,cc,cg,ct,ga,gc,gg,gt,ta,tc,tg,tt (16 features)	product dinucleotide frequencies
r7,r6,r5,r4,r3,r2,r1,s0,f1,f2,f3,f4,f5,f6,and f7 (15 features)	read abundance 7 nt downstrem to 7 nt upstream product start position
WFC	Wooton-Federhen Complexity of product sequence
Duplex Energy	Minimum free energy of product duplex with surrounding genomic region.

**Table 2:** Features used in the hairpin products random forest (MRPF).

Name	Description	Reference
mfe	minimum free energy of hairpin fold	14, 11*, 13*, 4, 10, 1, 12
pbp	frequency of paired bases of miR	11, 4*, 8
urf	fraction of unique reads to total adjusted reads for locus	34, 8
gcContent	GC content of locus sequence	11, 10
totalSenseRPM	Adjusted reads per million (ARPM) in the sense strand	12*, 4*
loopSize	length of the loop in nucleotides.	10, 12
maxBulge	longest bulge appearing in the region of the hairpin spanning the miR and miR*	10*, 12
tapd	total displacement of sense to anti-sense products	8
aapd	average displacement of sense to anti-sense products	8
ahc	average number of hits to the genome for the major product	8
afh	average 5’-heterogeneity of major product reads	8
sameShift	Amount of offset between products on the same arm	8
bothShift	maximum amount two products are offset on opposite arms	8
Dinucleotide frequencies (16 features)	precursor dinucleotide frequencies	12
maxInteriorLoop	Length of largest interior loop spanning the miR and miR*	12
intLoopSideDiff	Difference in length of of interior loop branches in miR/miR*	12
OPA	Frequency of the most abundant overlapping product	
Duplex Energy	Duplex energy of major product and surrounding region.	
foldDupCmp	Similarity between dotbracket sequences from RNAduplex and RNAfold	
dupPBP	base pairing density of region duplexing the major product	
dupLoopLength	Length of biggest bulge or interior loop in region duplexing the major product	
APV	The average variance of read counts for distinct reads for all products	
wAPV	The average variance of read counts for distinct reads weighted across products	
ARV	The average variance of start positions for reads on each product	
wARV	The average variance of start positions for reads weighted by product size	
mpLoopDistance	distance of the miR from the loop	
dupLoopDistance	distance of the miR* from the loop	
totalOverlap	The sum of the amounts of overlap between each pair of overlapping reads.	
totalRelativeOverlapAmount	sum of each overlap multiplied by the abundance ratio of the smaller to larger product	
averageOverlapAmount	sum of each overlapping product multiplied by the frequency of reads of the smaller product within the hairpin	
innerLoopGapCount	number of times 3 or more unbound nucleotides appears in the loop region	
totalAntisenseRPM	Adjusted reads per million (ARPM) in the anti-sense strand	
maxUnboundOverhang	The largest length of unpaired nucleotides on either side of the miR	
numOffshoots	number of additional hairpins formed on or across from the miR or miR*	
dupSize	The size of the region duplexed by the miR product	
neighborCount	The number of regions of contiguous read loci within 1000 nucleotides of the precursor	
RFProductAvg	Decision value returned by the random forest in the product phase	
Product counts (8 features)	The fraction of the product relative to the total for the hairpin	
Product overlaps (11 features)	Overlaping lengths for individual products within the locus (e.g. “miRmoR5pOverlap” the overlap between miR and moR on 5′ arm).	

*References with an asterisk use a variant of the described feature.

### Stacked random forest approach

As with other microRNA discovery tools, miRWoods begins by analyzing genomic loci where small RNA reads mapped. A distinguishing feature of miRWoods is the use of an additional RF layer (the MRPF) to classify reads stacks as plausible mature microRNAs rather than rely only on the number of reads mapping to that genomic locus. The features used in the MRPF are summarized in Table 1. The MRPF also leverages basic sequence features previously shown to be effective in detecting precursors such as GC-content and dinucleotide frequencies [10–14]. In addition, we introduce some novel features such as the duplex energy between the read stack’s most frequent read sequence and the surrounding genomic locus. This quantity is distinct from miR:miR* duplex energy because the input is a read stack, and miR/miR* designations have not been assigned at this point. Also included are the observed frequencies of 5′ read ends relative to the most abundant position.

The HRPF also uses several novel features, summarized in Table 2. Novel features include 11 “overlap” features, correpsonding to the degree of overlap between different identified products (e.g. 5’ moR, 5’ miR, loop, 3’ miR). We also introduced several features describing destabilizing structures, such as bulges and loops, and several features describing the regions duplexed with the most abundant product. We also analyzed what features were most important for miRWoods, and summarized feature importance in S1 Fig. We found that the frequency of reads in the start position of the read stack and the duplex energy to be highest in importance for the MPRF (S1A Fig). We found that the decision value from the MPRF, the reads per million in the sense and anti-sense strands, the product base pairing, and the duplex energy to be the most important features for the HPRF (S1B Fig). Because the value of some features showed correlation, we also examined feature importance for RFs trained with correlated features removed. We identified features with an R^2 greater than or equal to 0.5 (S1 Table), and removed the feature with the highest importance for each correlated pair. We did see an increase in feature importance for some features in the HRPF, such as totalSenseRPM, dupLoopDistance, ARV, wARV, dupPBP, and afh (S2 Fig). We also examined the change in importance when correlated features are removed (S3A and S3B Fig). In some cases, features gained the importance after removal of their correlated partner. Other cases, such as “dupLoopDistance” and “dupPBP” showed a substantial increase in importance despite not having correlated features removed. We do not observe a significant decrease or consistent change in performance with the most correlated features removed (S3C Fig).

We examined the role of read-abundance on the performance of miRWoods. The histograms of true positive predictions from miRWoods, miRDeep2, and miREAP demonstrate that miRWoods correctly identifies more single-read miRs (S4A–S4F Fig). We observed that consistently in both predictions trained and tested in human (same-species) and trained on human, tested on mouse (cross-species), miRWoods consistently makes more valid positive predictions for loci supported by only one read (S4G Fig). While these predictions illustrate the power of miRWoods, in practice any single-read predictions are not proof and would require further validation. We analyzed effect of removing read-abundance-related features and found that while performance does reduce with the removal of these features (S4H Fig), the overall greater performance on low-abundance loci demonstrates that these features do not impair performance.

### Accurate mapping of hairpin precursor span

**Fig 2:**
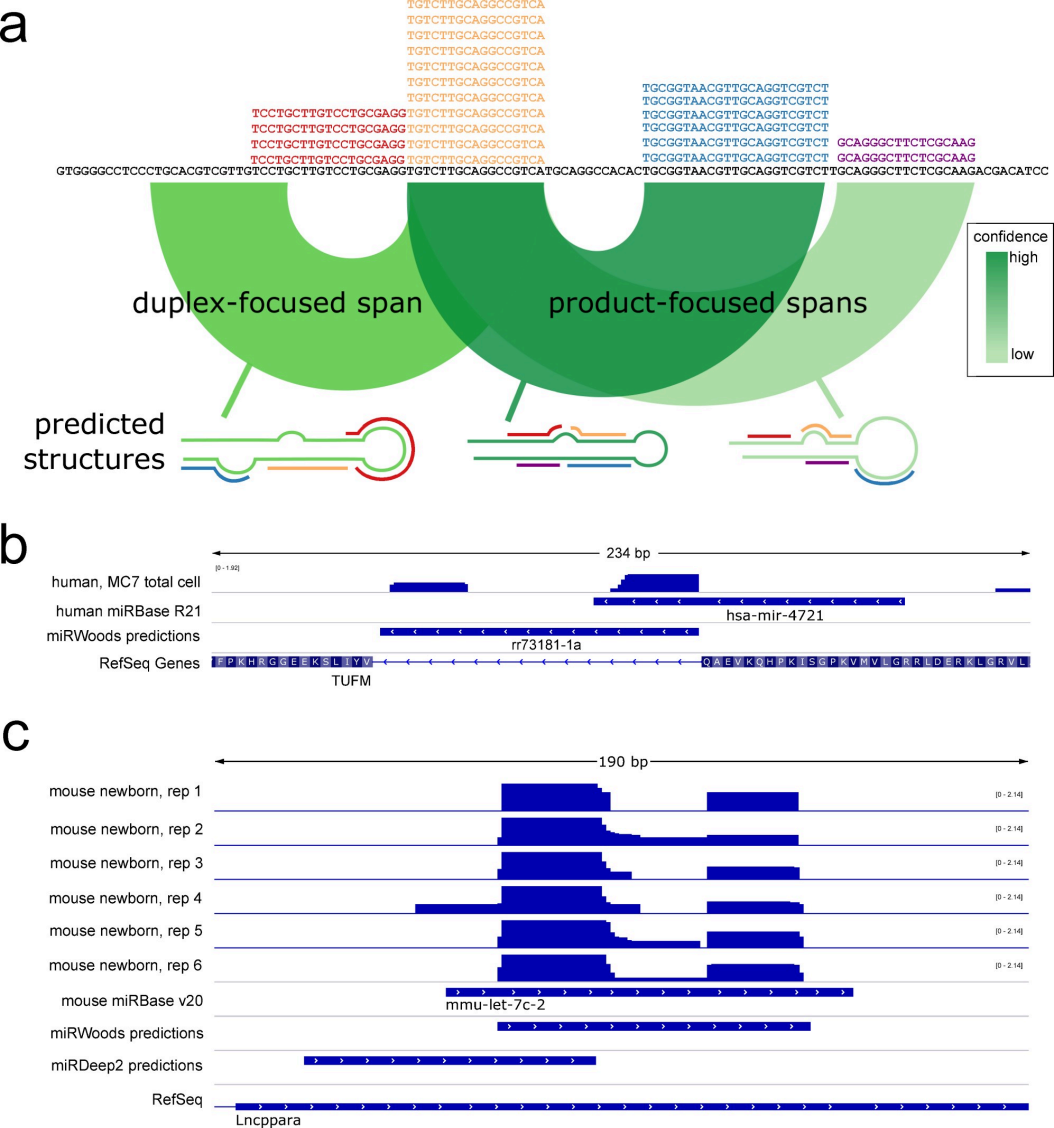
Improved hairpin precursor Span identification. **a** miRWoods generates several potential hairpin precursor spans from each product that passes through the MPRF. Duplex-focused spans take the region between the product and the optimal duplex and product-focused spans take the region between the product and other products greater than 4 nt away. Hairpins are selected based on HPRF score. **b** The miRBase annotation for hsa-mir-4721 crosses over an intron boundary. miRWoods corrects the annotation by recognizing a second read stack and produce precursor span that perfectly matches an intron, suggesting mir-4721 is a mirtron. **c** The miRWoods prediction for mmu-let-7c-2 in mouse is consistent with the miRBase annotation, while the best miRDeep2 prediction, albeit below the default signal-to-noise threshold, only partially overlap with the miRBase annotation.

Proper identification of hairpin precursor span is critical for microRNA prediction, because methods typically rely on secondary structure prediction, which can significantly depend on the defined window. The labeling of 5′ vs 3′ products requires accurate identification of the hairpin precursor. We imposed stringent requirements for predictions for the hairpin span of a locus to be considered a true positive when compared to miRBase annotations. Predicted loci where the hairpin folded in the wrong direction and/or overlapped less than 50% of the annotation were counted as false predictions. To address these stringent criteria, we developed an approach that focuses on strong miR/miR* duplex energy, rather than secondary structure of the hairpin. While most approaches focus on the predicted structure in a region around the most abundant product (i.e. major product), our duplex-focused method selects the span of hairpin regions using the optimal duplex pairing with the most abundant product (Fig 2A). Alternatively, product-focused spans covering the major product and any product 4 nt or more away from the major product are also considered. Each of these options are considered as putative loci, and evaluated in subsequent steps. We found that miRWoods uses the duplex-focused span an average of 88.4% of the time in its final predictions for all human sets (S2 Table).

The percentage of predictions that matched an annotation well enough to be considered a valid hairpin precursor was computed for miRWoods, miRDeep2 and miReap and summarized in Table 3. miRWoods predictions used the proper fold an average of 99.1% of the time for human samples and 99.9% of the time for mouse samples. miReap was able to predict the proper fold 98.2% of the time for human and 97.8% of the time for mouse. miRDeep2 was able to predict the proper fold 98.9% of the time for Human and 97.7% of the time for mouse. In some examples, miRWoods corrects errors in the miRBase annotations. In Fig 2B we show the current annotation for hsa-mir-4721. While miRWoods predicts a hairpin precursor that directly matches with intron splice junctions (a mirtron), the miRBase annotation only overlaps one mature product. Similarly, S5A Fig shows hsa-mir-6860, which miRWoods predicts to be a half-mirtron and the current miRBase annotation does not. In both cases the miRWoods predicted hairpin span lines up with the intron splice site, even though miRWoods does not use splice junction locations in its predictions, thereby providing independent support to the predictions. In other examples, such as mmu-let-7c-2, the miRDeep2 hairpin span is offset, assigning the 5′ product as the 3′ product (Fig 2C). A similar scenario is observed for hsa-mir-431 (S5B Fig).

**Table 3:** Percentage of predicted hairpin spans matching miRBase annotation. The method with the highest percent for a particular sample are presented in bold.

	miRWoods		miRDeep2		miReap	
library	total	percent (%)	total	percent (%)	total	percent (%)
human MCF-7 (total cell)	450	**98.901**	318	98.452	430	98.398
humam MCF-7 (cytoplasm)	452	98.69	314	**99.054**	428	97.717
human liver	385	**99.483**	318	99.375	413	98.804
human cell lines	736	**99.325**	532	98.519	228	97.854
mouse brain	405	**100**	330	98.214	370	98.143
mouse embryo	486	**99.59**	412	98.329	398	97.073
mouse newborn	419	**99.762**	335	97.384	179	97.283
mouse ovary	282	**100**	243	97.2	237	98.75
mouse testes	293	**100**	269	97.464	260	97.744

### Evaluation of prediction performance

A summary of all small RNA deep sequencing data sets is provided in S3 Table. The repertoire of expressed microRNAs can vary considerably between tissue types in the same organism; therefore, we tested miRWoods against different cell types and conditions. We tested miRWoods on 9 samples from 4 small RNA sequencing experiments and provide performance metrics compared to other methods in Table 4. We compared the performance of miRWoods, miRDeep2, and miReap on several small RNA data sets from human and mouse downloaded from GEO [15]. In each evaluation, the same RF models trained on human data were tested on small RNA data collected from different tissues including human MCF-7 total cell content (GSE31069), MCF-7 cytoplasmic fractions (GSE31069), human cancer cell lines (GSE16579), human normal liver (GSE21279), as well as cross-species tests on mouse brain, embryo, testes, ovary, and whole newborns (GSE20384). Because microRNA expression can vary from tissue to tissue, all programs were evaluated against the expressed miRs for that data set with at least one read aligned. miRWoods recalled on average 10% more annotated miRs, and obtained greater F-scores except in the case of mouse embryo where the F-sore was 0.312 for miRWoods compared to 0.313 for miRDeep2. Higher F-scores were obtained for all sets when miRWoods was compared with miREAP.

**Table 4:** Comparison of performance of miRWoods compared to miRDeep2 and miReap. The method associated with the highest F-score for a particular sample are presented in bold.

	miRWoods			miRDeep2			miReap		
library	precision	recall	F-score	precision	recall	F-score	precision	recall	F-score
human MCF-7 (total cell)	0.727	0.501	**0.296**	0.839	0.354	0.249	0.42	0.478	0.223
human MCF-7 (cytoplasm)	0.7	0.511	**0.295**	0.86	0.355	0.251	0.476	0.484	0.24
human liver	0.871	0.447	**0.295**	0.898	0.369	0.262	0.446	0.48	0.231
human cell lines	0.627	0.586	**0.303**	0.834	0.424	0.281	0.264	0.182	0.108
mouse brain	0.849	0.569	**0.341**	0.951	0.463	0.312	0.397	0.52	0.225
mouse embryo	0.694	0.567	0.312	0.898	0.481	**0.313**	0.205	0.464	0.142
mouse newborn	0.836	0.559	**0.335**	0.931	0.447	0.302	0.312	0.239	0.135
mouse ovary	0.953	0.603	**0.369**	0.96	0.519	0.337	0.798	0.506	0.31
mouse testes	0.91	0.579	**0.354**	0.944	0.532	0.34	0.324	0.514	0.199

**Fig 3:**
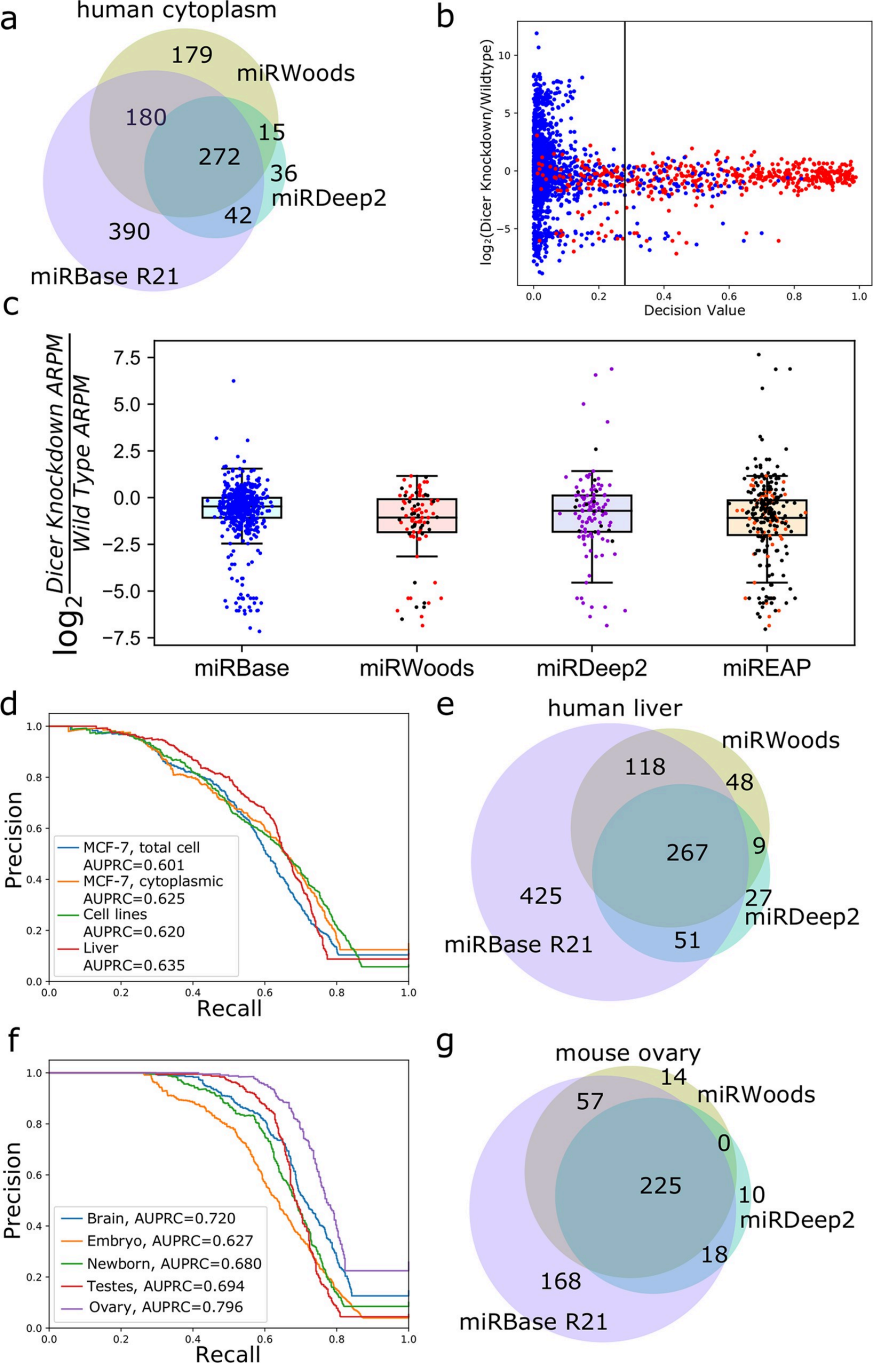
Evaluation of miRWoods performance. **a** Euler diagrams comparing predictions from miRWoods and miRDeep with annotations from miRBase for human MCF-7 cytoplasmic extract **b** A scatterplot comparing the miRWoods decision value to the log fold change in Dicer knockdown cells compared to wild-type cells. **c** Scatter-boxplot comparing the log fold change for Dicer knockout to wild type for unprocessed read regions, miRBase annotations, and predictions from miRWoods, miRDeep, and miReap for MCF-7 (cytoplasmic fraction). Black dots indicate predictions that are unique to this method. **d** Precision-recall (PR) Curve and AUPRC of miRWoods predictions for human including MCF-7 (total cell content), MCF-7 (cytoplasmic fraction), cell lines, and liver. **e** Euler Diagrams comparing predictions from miRWoods and miRDeep with annotations from miRBase for human liver. **f** Precision Recall Curve and AUPRC of miRWoods predictions for mouse tissues including brain, embryo, newborn, testes, and ovaries sets. **g** Euler Diagrams comparing predictions from miRWoods and miRDeep2 with annotations from miRBase for mouse ovary.

For a detailed summary of all microRNAs evaluated, both novel and annotated, along with expression levels across all samples, see S4 Table. Remarkably, miRWoods performed better on cross-species tests on mouse data compared to tests on human data (S6 Fig), providing justification for its application to other mammalian genomes when trained on human. Typically, miRWoods has a greater number of false positives and fewer false negatives than miRDeep2 when compared to miRBase annotations (Fig 3A).

We tuned thresholds for expression level, proportion of negative samples, and decision values threshold on a separate dataset from what RFs were trained on (see Methods, S7 Fig). A summary of the data sets and values resulting from the tuning experiment is provided in S5 Table.

The decision value threshold that has been tuned to optimize the F-score for the identification of valid loci correlates well with decreased expression in Dicer knockdown MCF-7 cells (Fig 3B, S8A and S8B Fig). On average, the novel predictions of miRWoods show a greater decrease in the cytoplasm of Dicer knockdowns compared to novel predictions from miRDeep2 and miREAP (Fig 3C) and on par in total cell content (S8B Fig). We calculated p-values for each of these comparisons using two-sample t-tests and found that novel predictions in cytoplasm from miRWoods and miREAP had a significant reduction in Dicer-knockdown expression compared to miRBase. Novel predictions in total cell content for all programs showed a significant reduction in Dicer-knockdown expression compared to miRBase (S6 Table). Similarly, empirical cumulative distribution functions (ECDFs) of the fold change in Dicer knockdowns compared to wild type show a greater proportion of novel predictions highly depleted in Dicer knockdowns (S8C and S8D Fig). Examples of novel predictions found to be reduced in expression in Dicer mutants include hsa-Novel35, hsa-Novel28, hsa-Novel23, hsa-Novel65, has-Novel92, and hsa-Novel99 (S9 Fig).

One advantage of miRWoods over the other methods is that it prints a score for each genomic locus evaluated, whether or not it is predicted to be a microRNA. Therefore, the output is amenable to creating precision-recall (PR) curves [16], such as Fig 3D and 3F. The area under the PR curve (AURPC) evaluates the performance of the prediction, and has the advantage over Receiver Operator Characteristic (ROC) curves [17] of not being overwhelmed by the large number of true negatives associated with genome-wide microRNA prediction. We present PR curves for predictions in mouse, with an average AUPRC of 70.3. Comparisons with miRDeep2 show that miRWoods has greater false positives, but fewer false negatives (Table 4, Fig 3E–3G, S10 Fig). Comparisons with miREAP show that miRWoods has a lower false positive rate, and a higher F-score on average (Table 4, S11 Fig). Overall, miRWoods shows equal or greater F-score than both miRDeep2 and miReap for all data sets (Table 4).

Many of the “false positive” microRNA predictions are actually novel predictions of valid miRs. Despite how complete the human microRNA annotation is, we were able to identify 682 potential novel loci in the human data sets. We found that many of our novel predictions, despite not being annotated, had homology to known miRs in other species. In some cases, miRWoods identified more instances of known miR families. For example, there are 72 known precursors from the mir-548 family in the human genome annotated by miRBase. miRWoods was able to identify an additional 34 novel members of the mir-548 family (S12 Fig), suggesting this family could be larger than previously thought.

### Novel microRNA predictions in the feline genome

**Fig 4:**
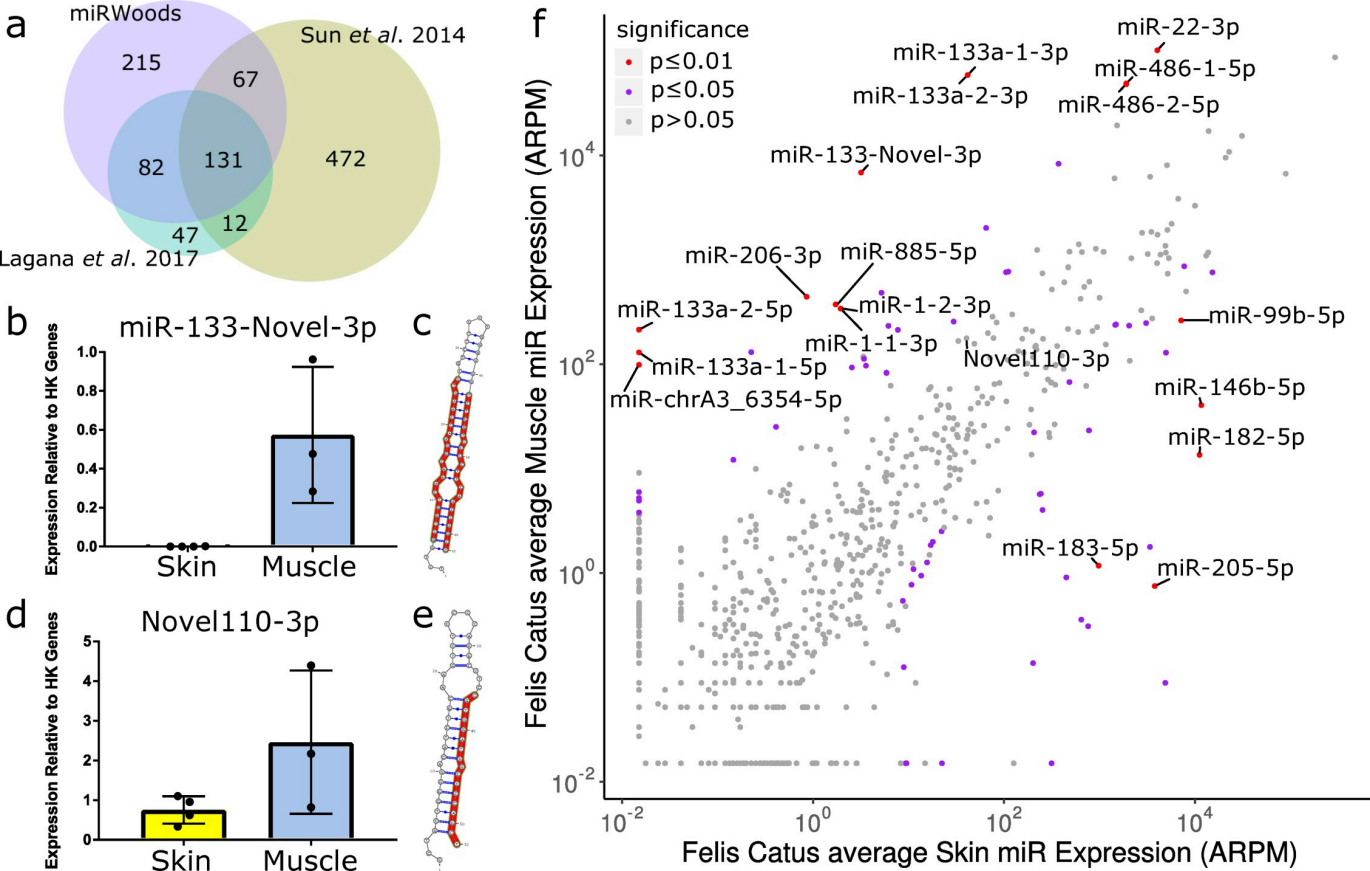
miRWoods predictions in the feline genome. **a** Euler diagram of the predictions from miRWoods with predictions from Sun *et al*. (2014) and Lagana *et al*. (2017). **b** The expression in skin and muscle for miR-133-Novel-3p **c** Hairpin for mir-133-Novel precursor. **d** The expression in skin and muscle for Novel110-3p. **e** Hairpin for Novel110 precursor. **f** Scatterplot of average muscle expression vs average skin expression for each mature microRNA.

We next sought to predict microRNA loci in species with limited microRNA annotations, including the feline and bovine genomes. We ran miRWoods on small RNA samples isolated from muscle and skin tissue for 3 different cats. Currently, there are two studies of feline microRNAs that we are aware of. In one study, Sun *et al*. did an analysis with miREAP in the context of the mink enteritis virus (MEV) [18]. In a more recent study, Laganà *et al*. identified feline microRNAs with miRDeep2 in a multi-tissue cohort [19]. miRWoods identified 495 microRNA loci, with 293 of them having significant homology to microRNA precursors from miRBase. Among the miRWoods predictions, 198 overlapped with the microRNA found in Sun *et al*., and 213 overlapped with microRNAs found in Laganà *et al*., and 215 were newly discovered (Fig 4A).

Expression of three novel microRNAs in feline skin and muscle were examined by qPCR and normalized expression relative to 2 control miRs with low variability across our tissue samples, miR-25 and miR-191 (Fig 4B and 4D, S13 Fig). These examples included a novel member of the miR-133 family, with enriched expression in muscle that was validated by qPCR (Fig 4B) and a predicted structure that strongly matches expectations for microRNAs (Fig 4C). We also identified a novel miR with no homology to known miRs, with a statistically significant tissue-specific enrichment based on a voom analysis [20], including some more abundant in muscle (Fig 4B–4E). In addition, we validated two predicted miRs previously described by Laganà *et al* that we determined to be significantly differentially expressed. As predicted, fca-mir-1-1 was more abundant in muscle whereas fca-mir-205 was abundant in skin (S13 Fig). Overall, our analysis of the expression of our predicted microRNAs identified 71 differentially expressed miRs using a voom FDR of 0.05, with 33 enriched in muscle, and 38 enriched in skin tissue.

**Fig 5:**
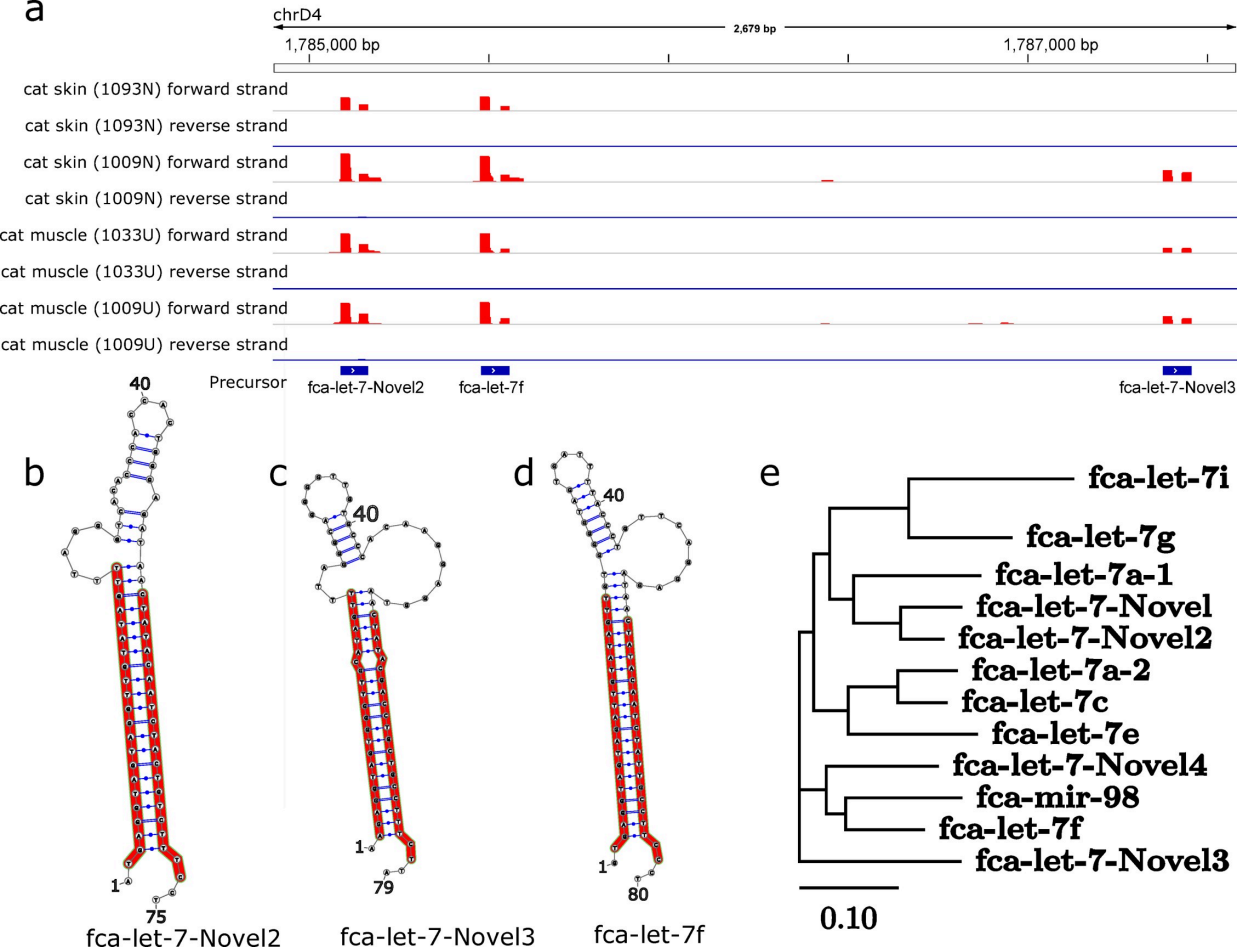
Novel let-7 microRNAs in the feline genome. **a** RNA-seq of cluster containing fca-let7-Novel2, fca-let7f, and fca-let7-Novel3 for each skin and muscle sample from *Felis catus*. **b** Hairpin structures for fca-let7-Novel2, **c** fca-let7-Novel3, and **d** fca-let7f. **e** Phylogenetic tree of let-7 miRs including those previously found by Lagana *et al*. (2017).

Several known and novel let-7 family precursors were found within clusters including multiple let-7 miRs. For example, we found a cluster on chromosome D4 containing fca-let-7f and two novel let-7 miRs denoted fca-let-7-Novel2 and fca-let-7-Novel3 (Fig 5A). The predicted novel miRs (Fig 5B and 5C) have predicted secondary structures with similar bulges and/or internal loops observed in other let-7 family members including fca-let7f (Fig 5D). A phylogenetic tree of known and novel let-7 miRs shows comparable sequence similarity, although not necessarily correlated with proximity of the genomic loci (Fig 5E).

Feline microRNAs were found within 51 clusters, 28 overlapped with the 31 previously described [19]. In addition to the two previously-identified feline-specific precursors within cluster 14, miRWoods identified one additional feline-specific precursor tagged as fca-Novel45. Cluster 2 covers an intron within the ARHGEF10L gene and contains two more novel feline-specific miRs (fca-Novel10 and fca-Novel13.) Cluster 3 contains fca-mir-30c-1, and two novel mir-30 homologs antisense to one another within an intron on the NFYC gene (S7 Table)

### Novel microRNA predictions in the bovine genome

For the bovine genome, there are 811 known microRNA precursors producing 881 mature microRNA annotations, compared to 1187 precursors for mouse and 1881 for human, which generate 2045 and 2813 mature products, respectively.

We used miRWoods to predict bovine microRNA loci using small RNA-seq samples from 10 bovine tissues including corium from the hoof (corium feet), dental pulp, oral papillae, penis, retina, iris, optic nerve, brain stem, bone marrow, and submandibular lymph node. We selected tissues that were highly diverse and whose microRNA profiles had not been examined before. Our pipeline identified a set of 810 predicted microRNA loci. Among these, 409 were already in the miRBase R21 *Bos taurus* annotations, 91 had homology to microRNA annotations in cow and other species, and 310 were novel predictions with no known homology.

Overall, miRWoods identified 401 novel bovine microRNAs. In addition, clustering of microRNA loci revealed 76 clusters, including 63 known and 13 novel clusters (S7 Table). Cluster 19 contained two bovine-specific half-mirtrons, (bta-Novel68 and bta-Novel71), within the *PLD2* gene. A bovine specific mirtron (bta-Novel210) and another half-mirtron (bta-Novel212) were found within the *MCAM* gene on cluster 53. Another bovine specific half-mirtron (bta-Novel208) was found on cluster 44 with bta-mir-140 on the *WWP2* gene.

**Fig 6:**
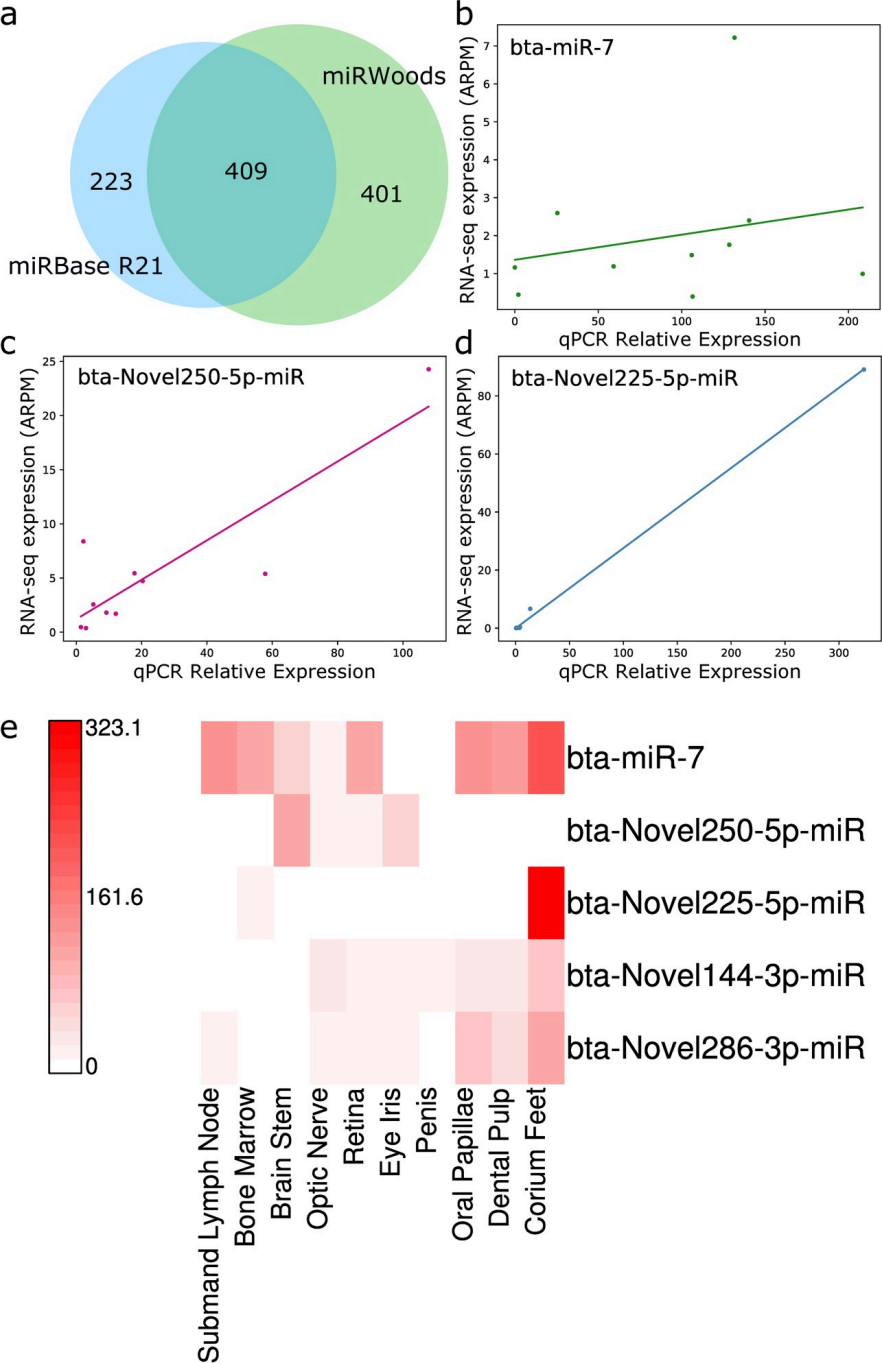
Novel microRNA predictions in the bovine genome. **a** Euler diagram comparing miRWoods predictions in the cow genome with miRBase annotations. **b** Scatterplot and best fit line comparing the normalized RT-qPCR expression and RNA-seq for the control miR bta-miR-7. **c** Scatterplot and best fit line comparing the normalized RT-qPCR expression and RNA-seq for a novel predicted miR with enriched expression in brain stem. **d** Scatterplot and best fit line comparing the normalized RT-qPCR expression and RNA-seq for a novel predicted miR with enriched expression in corium feet. **e** Heat map of RT-qPCR expression expression values over tissues examined.

Fig 6A shows an Euler diagram comparing miRBase annotations to miRWoods predictions for bovine samples. To test the validity of the novel predictions, we performed RT-qPCR on available samples, and normalized expression relative to 5 control miRs with low variability across our tissue samples. After normalization, expression levels for control miR-7 are compared with RT-qPCR (Fig 6B). Strong correspondence between small RNA-seq and RT-qPCR are observed for 2 of the tested microRNAs (Fig 6C and 6D), suggesting that the mature product was detectable with both methods in the tissue it was expressed. Expression was observed for all tested novel bovine miRs using RT-qPCR, validating the expression of these predicted mature products (Fig 6E).

### Novel Predictions in the Bovine miR-2284 Precursor Family

**Fig 7:**
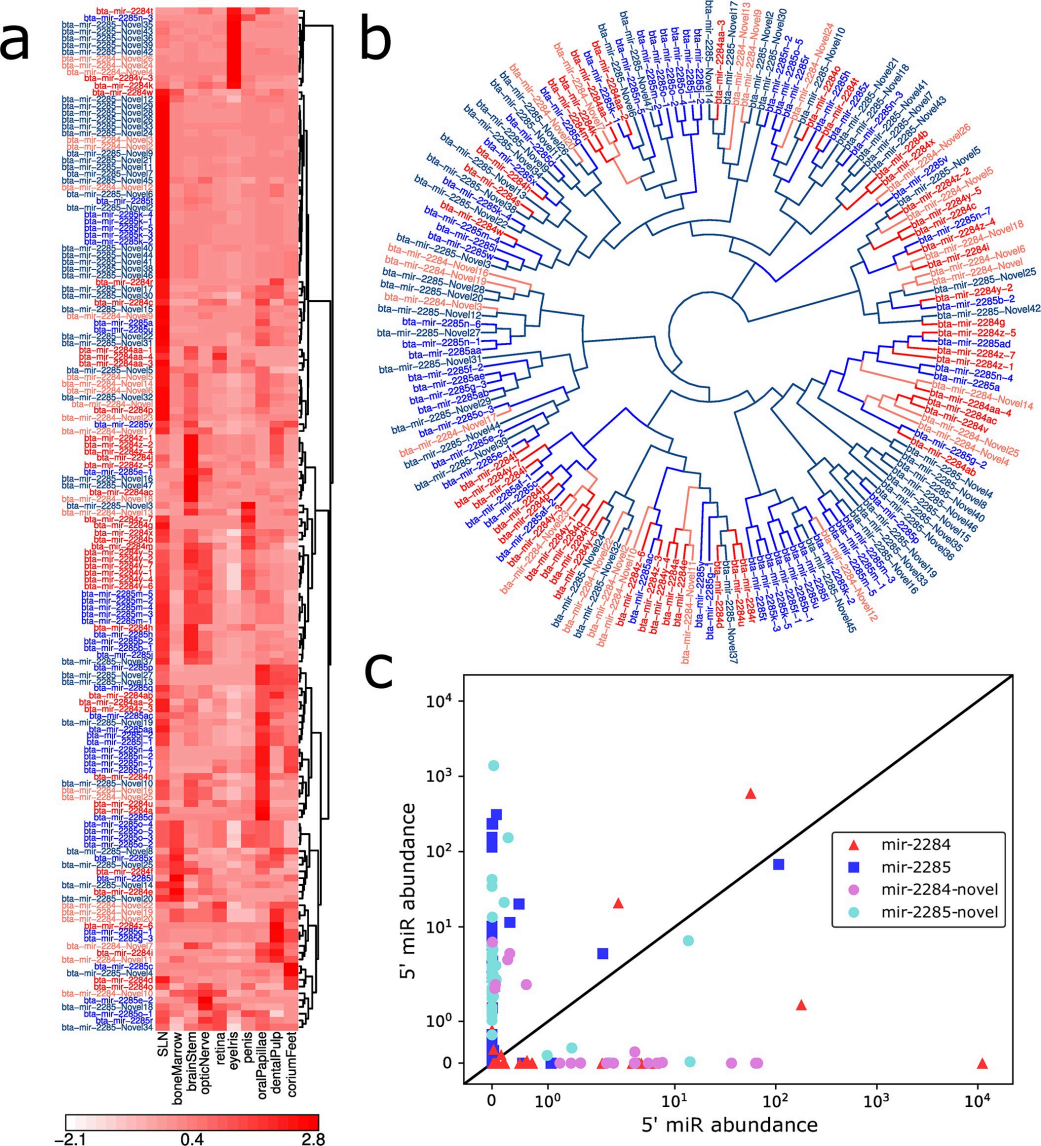
mir-2284/mir-2285 family miRs in *Bos taurus*. **a** A heat map for the expression of annotated and novel mir-2284/mir-2285 family miRs. **b** A phylogenetic tree for the bta-2284/bta-2285 family. Variants of bta-mir-2284 appear in red and variants of bta-mir-2285 appear in blue. Colors for novel predictions appear lighter than those for annotated predictions. **c** Abundance of miRs for the 5′ and 3′ sides of the mir-2284/mir-2285 family. The 5′ product tends to show greater expression in the mir-2284 family while the 3′ product shows greater expression in the mir-2285 family.

The miR-2284 family has previously been found to be expressed in tissues relevant to the immune system but gene targets are currently unknown [21]. Within the mir-2284 family, miRWoods predicted 29 known and 68 homologous precursors. Of the 68 homologous precursors, only 35 fit the criteria of having the same seed region as other miRs. Removing the seed requirement, 33 additional mir-2284 family precursors were identified. Unique reads were found in 51.5% of homologous precursors and 37.00% of already annotated precursors. Hierarchical clustering was performed on mir-2284 family microRNAs based on their normalized expression profiles and a heat map was generated (Fig 7A). Despite having a shared homology, expression of the microRNAs in the mir-2284 family are highly diverse in the tissues assayed, but show greatest expression in submandibular lymph node (SLN). Interestingly, this is consistent with prior studies of this family that observe greatest expression in bovine immune cells [22] given recent studies of the immunosuppressive properties of SLNs [23]. A phylogenetic tree was created to show all annotated and newly predicted miRs in the mir-2284 family (Fig 7B).

Read abundances showed a tendency for mir-2284 and mir-2285 precursors to favor a single (opposite) side of the precursor. The abundance of each microRNA for the mir-2284 and mir-2285 precursors within the mir-2284 precursor family was examined (Fig 7C) For annotated microRNA precursors, 82.05% of mir-2284 loci had the most abundant read on the 5p-side, and 89.36% of mir-2285 had the most abundant read on the 3p-side. Similarly, for our predicted microRNAs with homology to this family, 73.91% of mir-2284 examples had the most abundant read on the 5p-side, and 88.89% of mir-2285 examples had the most abundant read on the 3p-side.

### Discovery of novel miR families

**Fig 8:**
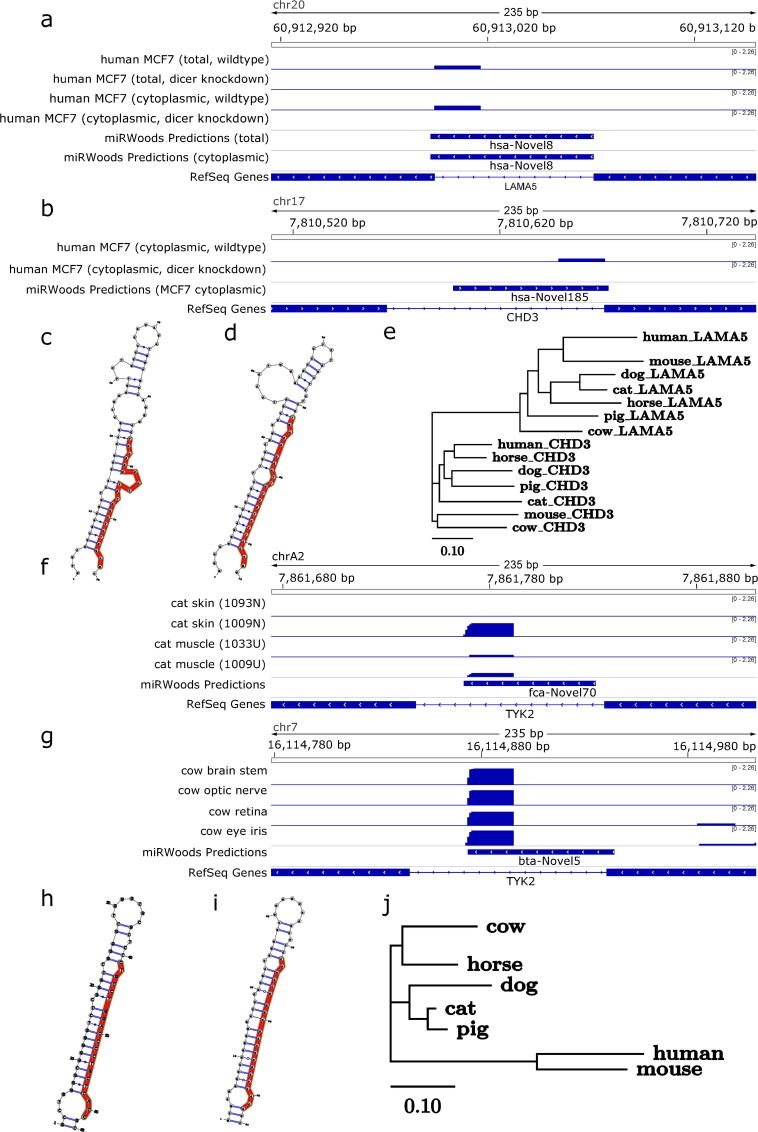
Novel microRNA families identified by miRWoods. **a** hsa-novel-8 is a mirtron predicted for both MCF-7 sets where expression was decreased in the Dicer knockdown sets. **b** hsa-Novel-185 is a mirtron predicted within the human cell lines set and the MCF-7 (cytoplasmic fraction) set. It also shows reduced expression in the Dicer knockdown version of the MCF-7 set. **c** The structure of hsa-novel-8. **d** The structure of hsa-Novel-185. e Phylogeny comparing the LAMA5 intron and CHD3 intron for several mammals. **f** Novel miR predicted in bovine genome in an intron of TYK2. **g** novel predicted miR in the feline genome in the same intron of TYK2 **h** structure of novel feline miR. **i** structure of novel bovine miR. Eight nucleoties were removed from the 5' end, and two were added to the 3' end to match the feline hairpin precursor boundaries. **j** A phylogeny comparing the TYK2 intron in several mammals.

We found that 11 novel predictions in human were within clusters of annotated microRNAs, and 39 novel predictions in new clusters. Of the 9 potential miR families which matched the criteria found in the methods section one contained a snoRNA and was removed. Of the remaining candidate miR families two had miRs which were found across species. We identified a novel miR family with two instances in the human genome; one example was a mirtron in an intron of *LAMA5*, and the other a half-mirtron in an intron of *CHD3* (Fig 8A–8E). Both of the examples in human were observed to have no expression in Dicer knock-out cells (Fig 8A and 8B). We observed a strong level of similarity in predicted secondary structures of the two examples observed in human (Fig 8C and 8D). We compared these introns across several mammalian species and observed patterns of conservation that suggest an ancestral divergence of these two mirtrons rather than a more recent duplication (Fig 8E). We found another novel miR family with an example in both the bovine and feline genomes, but not observed in mouse or human (Fig 8F–8G). Strikingly, both of these miRs (bta-Novel5 and fca-Novel70) were found within the same intron of *TYK2* in cow and cat genomes (Fig 8F and 8G), and both examples showed nearly identical hairpin precursor sequences (Fig 8H and 8I). We did not observe this miR in human or mouse data sets, and we also observed greater sequence divergence of this intron in human and mouse (Fig 8J). A list of all identified putative novel families identified by miRWoods is presented in S8 Table.

## Discussion

Our study demonstrates that despite a long history of microRNA discovery tools and annotations, there is still room for improvement. Despite the maturity of microRNA annotations for the human genome, our approach was still able to find novel human miRs. We have identified several miRs with annotated positions shifted from the correct location, and that have been resolved with miRWoods.

The inclusion of the duplex-focused method in miRWoods improved hairpin precursor span identification over the other programs. Not only did miRWoods match the miRBase hairpin precursor annotation more often, in some instances miRWoods predictions corrected the miRBase annotation. Splice junction boundaries for the mirtron and half-mirtron examples provide evidence for the validity of the miRWoods duplex method because the optimal precursor span closely corresponds to the splice junctions, as expected given mirtron biogenesis mechanisms [10] despite the fact that these hairpin boundaries were computed without the use of intron annotations. Similarly, Boruta feature-importance analysis showed that the duplex energy was more important than the minimum free energy of the hairpin. These observations support the idea that the thermodynamic stability of intermediate RNA duplex formed by miR and miR* may serve important roles in microRNA function, consistent with previous studies showing this affects efficient loading into Argonaut [24]. We also found that the distance between the miR* sequence and the loop have a greater importance than that of the major product and the loop. Future work is needed to determine the relative importance of stable mature miR:miR* duplex formation compared to stable stem-loop formation in microRNA biogenesis.

We demonstrated in this study that miRWoods is capable of correctly identifying microRNA loci with only one read more than other programs. Although this displays the strength the miRWoods approach, in practice users should seek further evidence to support the validity of any novel miRs only supported by one read.

Predictions from miRWoods consist of 215 potential novel microRNA annotations for cat and 417 novel candidates for cow. These findings support the expectation that these organisms have comparable number of microRNAs to human and mouse, but are currently less-well annotated due to greater research focus on human and mouse. Future work could expand miR annotation in feline and bovine further by sequencing other tissues, as well as identifying regulatory targets for miRs in specific tissues.

Finally, our approach is able to identify more examples of known families, suggesting that they are larger than previously thought. While these large families retain sequence similarity at the hairpin-level, they are often the result of seed shifting and mismatches, suggesting a wide range of potential gene targets. Predictions using miRWoods showed an expansion in the number of microRNAs within the mir-548 family in human, and the mir-2284 family in the bovine genome. These families are often defined in terms of homology to the hairpin sequence rather than the seed [25]. We observed several mutations within the seed region of mir-2284 family miRs that result in the complex phylogeny, and which indicate that a much wider range of genes may be targeted by this family than currently accepted. The fact that we observed miR-2284 family members to be differentially expressed across diverse tissue types supports the idea that this family expanded and sub-functionalized in various tissues. As noted previously, the widespread genomic distribution of the primate-specific mir-548 family supports the hypothesis that it may have been evolutionarily derived from transposable elements [26]. Similarly, mir-2284 family may be more expansive than previously thought, and the observed diversity of sequence and expression supports the hypothesis that this family has shaped ruminant evolution [25].

## Methods

### Ethics statement

All bovine tissues were harvested from animals that were already scheduled to be slaughtered, and collected immediately after slaughter. All slaughter operations were performed under USDA-FSIS supervision in accordance with the Humane Slaughter Act (1978), the Federal Meat Inspection Act (1906), and using a percussive captive bolt stunner. The feline tissue samples were obtained through the biobank at the Carlson College of Veterinary Medicine at Oregon State University. Tissues had been banked for research purposes with owner consent and approval of the institutional animal care and use committee.

### Tissue samples small RNA sequencing

We examined small RNA samples collected from 10 bovine tissues including submandibular lymph node (SLN), bone marrow, brain stem, optic nerve, retina and iris of the eye, penis (corpus cavernosum), oral papillae (buccal mucosa), dental pulp, and hoof corium (corium feet) from three Angus steers collected just after slaughter at the Meat Science laboratory at Oregon State University. The feline tissue samples were obtained through the biobank at the Carlson College of Veterinary Medicine at Oregon State University and included normal haired skin and normal skeletal muscle from three male neutered domestic short hair cats aged 10–13 years. Tissues had been banked for research purposes with owner consent and approval of the institutional animal care and use committee. RNA was isolated from tissue by chloroform-isopropanol extraction. RNA quality was analyzed on a Bioanalyzer 2100 Nano chip (Agilent Technologies, Santa Clara, CA), with a minimum acceptable RIN of 7. Small RNA sequencing was performed at the Center for Genome Research and Biocomputing (CGRB) at Oregon State University (OSU). Libraries were prepared using the Illumina TruSeq small RNA sample preparation kit (Illumina, San Diego, CA) for library preparation and size-separation by polyacrylamide gel electrophorese. Library size was determined with the Bioanalyzer 2100 HS-DNA chip and the KAPA biosystem’s library quantification kit, and libraries normalized to 2 nM. Multiplexed samples (6/lane) were sequenced with a 50 cycle v3 sequencing kit on an Illumina HiSeq 3000 sequencer.

### The miRWoods pipeline

The miRWoods pipeline consists of two random forests with readily interpretable, biochemically-motivated features. The pipeline’s two layers correspond to classifiers that recognize different components of the microRNA (Fig 1). The first random forest layer predicts likely mature miRNAs products. In this way, the first random forest acts to filter out a large number of loci before precursors are considered, thereby improving accuracy and reducing the overall runtime. The second random forest layer scores the precursors around the predicted mature miRNAs and is used to generate the final set of predictions.

The miRWoods pipeline is perl software largely derived from miRTRAP [4], but with significant improvements on speed and memory efficiency, as well as two random forest layers rather than user-defined thresholds. The pipeline now includes the integration of indexed bam files for faster read processing, and the RNAfold perl module for rapid secondary structure prediction. The processing of sequencing data begins with one or more small RNA-seq FASTQ files. We first trim the reads using cutadapt, which removes 3′ adapter sequences and filters for read quality requiring a PHRED score of 30 or greater [27]. Sequencing data is mapped to the genome using bowtie [28]. Before sorting and indexing the bam files, we add additional tags according to samtools specifications describing the number of hits (NH-tags) to the genome for each read [29], which is used later in the pipeline to normalize expression.

### Mature product random forest

The miRWoods pipeline consists of several data-processing steps. Next, after read alignment, we identify “read regions”, which consist of reads that map to overlapping positions in the genome. Each of these read regions are evaluated as putative mature microRNA products based on a number of features calculated from genomic loci and read distributions. Basic features for each read region are computed, such as GC-content, dinucleotide frequencies, Wootton-Federhen sequence complexity [30], and the median length of reads mapped to the locus. Because the function of microRNAs involves the position of seed sequences relative to the 5′ end, the 5′-heterogeneity is computed for each read region as previously described [31]. In addition, we compute the number of reads mapping to positions within a fixed offset from the most abundant product. We also computed the minimum duplex energy between the read stack’s most frequent read sequence and the surrounding 70bp region. These and other features are input into our first random forest, called the “mature product random forest” (MPRF), which classifies read regions as mature microRNA products or non-miR loci. For a complete list of computed features, see Supplementary Methods.

### Hairpin precursor span optimization

We found that a major source of error in high-throughput microRNA discovery was the prediction of the span (start and end positions) of genomic location of the hairpin precursor, and therefore we developed a new method of precursor span prediction (Fig 2). While most other approaches predict secondary structure of the region surrounding a putative mature product, our approach computes the RNA:RNA duplex energy of the mature products (without the loop). Each putative microRNA product identified by the MPRF is used to compute the optimal duplex energy between the most abundant product and the surrounding 70bp window using RNAduplex [10], as depicted in Fig 2A. The region spanning this most abundant read and the optimal duplex subsequence is then used as a putative hairpin precursor sequence. In addition, a second method folds between any two products that are separated by 5 nt or more. Both methods are used and create several secondary structure predictions, all of which are the basis of a putative hairpin precursor to be input to the next random forest. When the hairpins are subsequently evaluated in the next step, overlapping hairpins are dropped and only the predicted hairpin with the highest decision value from that random forest is retained.

### Hairpin precursor random forest

The second random forest within miRWoods, called the “hairpin precursor random forest” (HPRF) is used to evaluate the putative hairpin precursors from 71 features, which provide scores based on its sequence, structure, and folding energy. Many of the features for the hairpin phase come from the original miRTRAP software [4].

The features for the HPRF can be categorized as sequence features, structural features, and product-distribution features. Sequence features include dinucleotide frequencies, GC Content, and sequence complexity over the entire precursor sequence. Structural features include the minimum free energy returned by RNAfold [32], and the optimal duplex energy of the most abundant product and hairpin precursor region computed by RNAduplex [33]. The decision value from the MPRF for the most abundant product within putative hairpin precursors is also included as a feature.

Expression levels for a locus *L* are quantified with adjusted reads per million (ARPM), which are defined by

$$ARPM(L)=\frac{10^{6}\sum_{r\epsilon L}1/n_{r}}{\sum_{r\epsilon S}1/n_{r}}$$ (1)

Total read counts, separately computed for the sense and antisense strands of the precursor, are first adjusted, meaning when a read *r* aligns to *n*_*r*_ locations in the genome, the read contributes a fractional count of 1/*n*_*r*_ to each location, essentially uniformly distributing the count to each locus [34]. These calculated values are then normalized for each sample *S* to parts-per-million.

The product-distribution features are computed by first naming read stacks as the products that would be expected in the event of Dicer and Drosha cuts by a previously defined algorithm [4]. A number of features describe the abundance and mapping of reads for each of these products. The unique read fraction describes the proportion of reads mapping only to the locus. Various features, such as the 5′ heterogeneity, and average hit count were evaluated for the most abundant mature product. For each of the mature products, several features describe the relative frequency of reads for miRs, moRs, loop products, and other products within the precursor. Several other features were created to describe the variance and weighted variance of reads associated with mature products relative to the most frequent cut variant and to the hairpin.

Dicer and Drosha tend to make precise cuts to produce well-defined 5′ ends of the mature products for proper functionality. Because of this, several features describe the amount of overlap across all products and across each product relative to its surrounding products. In addition, reads within a product would not be expected to be significantly offset from the product on the opposite arm of the hairpin, or relative to any moR products on the same arm. Therefore, features measuring the amount of shift between miR products are included.

A number of features were generated to describe the structure of the predicted hairpin. Two features, base pair density (fraction of paired nucleotides in predicted structure) within the major product, and base pair density within the optimal duplexed region. These features may be different due to bulges being present on one arm of the hairpin but not the other. Features for the part of the fold around the miR products include the sizes of the largest bulge, size of largest internal loop, size difference between the two halves of internal loops, and overhangs on the major miR product, which are defined as the maximum number of unpaired bases on either end of the miR. The dupLoopLength feature measures the largest region of unbound nucleotides on the duplex across from the most abundant miR Product. A dupSize feature is a measure of the size of the region predicted to duplex with the most abundant product. Since the duplex is expected to be around the same size as the miR product this feature may help exclude cases where there are large unpaired stretches on the duplex or most of the major product is unbound to the duplex. A feature called innerLoopGapCount scores the number of occurrence of spans of 3 or more unpaired nucleotides in the loop region (i.e. more than one indicates a multi-branched loop). This feature may help in situations in which there is a multiloop or where the loop structure is uncommon to known miR precursors. Additionally, a feature measuring the size of the hairpin loop is included. We added new features quantifying the size of the largest bulge in the hairpin structure, which is known to affect Dicer specificity [35].

Because microRNA loci tend to cluster together, we incorporated a neighbor count feature, which is a score tallying the number of neighboring hairpins that occur within 1000 nucleotides of the precursor being analyzed. The neighbor count feature counted all small RNA loci, including both miR and non-miR loci, and reduced the number of observed false positives.

### Training, tuning and model selection

The miRWoods pipeline requires models for both MPRF and HPRF layers that have been trained on positive examples, which are annotated microRNAs, and negative examples, which are loci containing read regions not overlapping annotated microRNAs. The training data for the MPRF is produced by a script that collects loci based on the overlap of the products with the mature microRNAs in miRBase annotations, with *X*-fold more negative examples than positives for some input *X*. The training data for HPRF is created by using hairpins with the best overlap of the known hairpin annotation.

Our strategy for tuning the thresholds of miRWoods focused on three parameters: the decision value ${\hat{y}}_{HPRF}$ for the hairpin random forest output, the expression level threshold *E*_*th*_ in units of ARPM, and the proportion *X* of negative loci used in stratified sampling. To determine these thresholds, we trained and tested on different small RNA deep sequencing data sets. We selected four large data sets from sequencing read archives (SRA) from diverse tissues and developmental stages. We trained on one of the data sets, which produced optimal RFs. We then applied this to a second data set and computed F-scores for different ${\hat{y}}_{HPRF}$, *E*_*th*_, and *X* parameters, and chose the set of parameters that gave the highest F-score.

Our strategy for training and tuning models was to train with one data set, tune on another, and ultimately select final models were chosen based on the highest F-Score when tested on a test set. Two sets of models were trained using either tonsillar B-cell populations from GSE23090 or human cerebellum, heart, kidney, and testis tissue from GSE40499 (S7A Fig). The frontal cortex data was excluded from the GSE40499 set to make read counts in tissues more balanced. Each of the two resulting models were tuned using a grid-search for the ${\hat{y}}_{HPRF}$, *E*_*th*_, and *X* parameters to optimize F-score when evaluated on either cancer cells from GSE18381 and GSE20592 or stem cells from GSE65706 and GSE62501; therefore, four tuning experiments were performed, corresponding to the four arrows in S7A Fig. Afterwards, models tuned using the cancer cell sets were validated using the stem cell sets and vice versa. The model resulting in the highest F-score from the test set was chosen for all remaining tests. Plots of the F-score as a function of each of the tuned parameters are presented in S7B–S7D Fig. In each training experiment the stratified sampling for the product model was set such that the negative set would be equal in size to the positive set. This was to allow as many products as possible to enter the hairpin phase while still filtering out enough that the resulting folds could be generated in reasonable amount of time.

The model with the highest F-score resulted from training on the set of tonsillar B-cell populations (GSE23090) and tuning on human melanoma cells (GSE18381) and human normal and cancerous cervical cells (GSE20592) when validated against stem cell sets (GSE65706). Tuning through a grid search resulted in an optimum decision value of 0.28, an ARPM of 0.11, and a 1:25 ratio of positive to negative training data used in stratified sampling.

### Comparisons with other tools

miRWoods was compared with miRDeep2 and miReap in the prediction of microRNA loci from small RNA sequence data in well-annotated genomes. The data used were MCF-7 cell cytoplasmic and total-cell extract from GSE31069, human cancer cell lines from GSE16579, healthy human liver samples from GSE21279, and mouse brain, embryo, newborn, testes, and ovary from GSE20384. For miRDeep2 the FASTQ files were combined and the program was run with the same settings as previously published [6].

We ran miReap with default parameters. FASTQ files were combined into a FASTA file with its reads collapsed. Reads were aligned with bowtie using the same settings used for miRWoods. However, because miRWoods uses quality scores and miReap does not, the allowable error outside of the bowtie alignment seed was changed from 50 to 80 to allow for at least 2 mismatches. Bowtie considers the default value of a mismatch without quality scores present to be 40 (see Supplementary Methods).

In order to provide a comparison of the three pipelines, a separate set of scripts was used to determine accuracy. For each pipeline being tested a common set of functions was used to score each prediction as a true positive or false positive. We imposed more stringent requirements for true positives than most previous studies that require just overlap with annotated microRNAs. Predicted hairpins where the precursor folded in the wrong direction and only partially overlapped the annotation were named “overlaps” and scored as false positives. Additionally, precursors on the antisense strand of an annotation were named false positives because there is uncertainty whether they are really active as miR precursors.

A set of custom-made scripts was also developed to find homology for novel predictions from each of the three pipelines. Mature products from precursors that did not overlap annotations were searched with BLAST to the database of mature microRNA found in miRBase [36]. Mature products were named homologous if they had the same seed region and an E-value less than 0.05 when compared with a miRNA in the database.

The sensitivity, specificity and F-scores were used to compare each of the three pipelines. The F-score was used to evaluate performance for two reasons. First, different mapping and filtering methods result in variable numbers of precursors being expressed. Because the F-score does not rely on a tally of the number of true negatives, it is better for comparisons. Second, the type of data being analyzed will tend to be very unbalanced with far more non-miRs than miRs, which leads to an inflated accuracy.

### Dicer knockdown comparisons

The differences in microRNA expression between wild-type cells and cells in which Dicer had been knocked down were compared across pipelines. Small RNA samples collected from total cell content and cytoplasmic fraction for this test came from the series GSE31069 downloaded from GEO. For each pipeline a set of predictions was generated for both wild-type samples. In each case, the log fold change was computed for each novel prediction comparing the expression of the wild-type cells versus cells in which Dicer had been knocked down. A pseudocount of 0.015 ARPM was used to avoid taking the log of zero.

### Validation of bovine and feline microRNA predictions

Novel microRNA predictions were evaluated with homology to known microRNAs from other species and validated by qPCR. We validated the expression of the novel miRs with the highest decision value using qPCR across the tissues we examined.

#### Feline microRNAs

Feline RNA samples were reverse transcribed with the HiSpec Buffer system of the miScript II RT kit. We performed qPCR in 96 well plates with the ABI StepOnePlus using cDNA generated from 2.5 ng total RNA, miScript Primer assays, and miScript SYBR Green PCR mix combined in 25 μL reaction volumes. Cycling followed manufacturer’s instructions. Melt Curve analysis was performed to insure single product generation and the average of all primer efficiencies was 1.8. Of the four potential reference genes selected from feline sequencing data, two, miR-25 and miR-191, were found to be stable across tissues and the average of CT values was used to normalize expression.

#### Bovine microRNAs

RT was performed using the miScript II RT Kit and qPCR was performed in a HT7900 ABI system in 384-well plate using the Custom miScript Primer Assay and miScript SYBR Green PCR Kit, following the manufacturer-instructions with a 4-fold dilution of cDNA prior qPCR. We performed normalization using internal control genes (ICGs) or reference genes as indicated by the MIQE guideline [37]. It has been proposed and demonstrated that the use of ICGs for normalization for miRs qPCR provides a more accurate measure of expression than other methods, such as normalization with 5S RNA, U6 snRNA, or total RNA [38]. In order to identify the best ICGs to normalize the novel miRs, we selected predicted miRs with low-variability and similar levels in expression across various tissues, as previously performed [38]. The miRs selected for bovine were miR-7, bta-miR-32, bta-miR494, bta-miR-1388, bta-miR-2431, bta-miR-2483, and bta-miR-6520; Final qPCR data for bovine were analyzed using LinRegPCR to account for efficiency of amplification [39]. Bovine qPCR data from the tested internal control miRs were normalized using geNorm to determine the M- and V-values [40]. bta-miR-7 had a M-value >1.5 and was therefore not used for normalization but rather as a positive control, while the most stable miR pair was miR-494 and miR-6520 (M = 0.98). The most stable normalization was obtained by using the 6 most stable miRs with a final V-value of 0.245. The normalization factor was calculated by geNorm as the geometrical mean of the most stable miRs.

### Hierarchical clustering

Hierarchical clustering was performed for the expression of known and novel mir-2284/mir-2285 family miRs in bovine. Expression was normalized by computing z-scores, subtracting the mean and dividing by the standard deviation across tissues.

### Identification of clusters

Clusters were identified by locating sets of precursors with genomic positions within 10 kbp of each other. Prior to detecting clusters using novel predictions, the set of annotated microRNAs were grouped into clusters. This was done first because if novel microRNAs fell within a cluster of annotated microRNAs it may count as further evidence that that microRNA is real. After the clusters of annotated miRs were identified, novel microRNAs were grouped into new clusters or incorporated into clusters of annotated microRNAs.

### Identification of novel miR families

In order to search for novel miR families, the sequences of each novel miR was blasted to a set containing a combination of the novel miRs found using miRWoods and the set of all known miRs from miRBase. Family membership requires a perfectly matching seed sequence, both products were on the same arm for each hairpin, and a BLAST E-value less than or equal to 0.5 for the mature product. In addition, we excluded examples with top hits that are antisense to itself and cases with identical mature sequences to prevent inclusion of loci originating from repetitive regions.

## Supporting information

S1 Methods Supplementary Methods. A document of providing additional detail for methods used for miRWoods.
(DOCX)

S1 Fig Importance of features. **a** The importance of each feature based on the Boruta analysis for the mature product random forest (MPRF) **b** The importance of each feature based on the Boruta analysis for the Hairpin Random Forest (HPRF).
(TIF)

S2 Fig Further feature interpretation: Removal of correlated features I. **a** Boruta analysis of feature importance for MRPF with correlated features removed. **b** Boruta analysis for HRPF with correlated features and the MRPF decision value removed.
(TIF)

S3 Fig Further feature interpretation: Removal of correlated features II. **a** Change in feature importance for MRPF with correlated features removed. **b** Change in feature importance for HRPF with correlated features removed.**c** the F1-score for miRWoods with correlated features and MRPF decision value removed compared to the full feature set.
(TIF)

S4 Fig Abundance-related features. **a** Distribution of read abundance for correct miRWoods predictions on MCF7 total cell content. **b** distribution of read abundance for correct miRDeep2 predictions on MCF7 total cell content. **c** distribution of read abundance for correct miReap predictions on MCF7 total cell content. **d** Distribution of read abundance for correct miRWoods predictions on mouse embryos. **e** distribution of read abundance for correct miRDeep2 predictions on mouse embryos. **f** distribution of read abundance for correct miReap predictions on mouse embryos. **g** bar plot of correct predictions with only one read for all samples in human and mouse. **h** F1-score of predictions with size-related features compared to without.
(TIF)

S5 Fig Effectiveness of duplex method. **a** RNAseq for hsa-miR-6860 shows miRWoods prediction covering an additional read stack next to the splice junction, which indicates that hsa-miR-6860 may be a half-mirtron. **b** RNAseq for hsa-mir-431 showing predicted folds for miRWoods and miRDeep.
(TIF)

S6 Fig Cross-species performance. Comparison between cross-species F1-score and same-species F1-score. All of miRWoods evaluations were tested on a single model trained and tuned on human datasets. The best performance is observed on mouse samples.
(TIF)

S7 Fig Tuning miRWoods. **a** Analysis pipelines and corresponding data sets used for training, tuning, and testing correspond to the paths of the arrows. **b** Plot of F1-score versus decision value threshold used in tuning the decision value threshold. **c** Plot of F1-score versus ARPM threshold used in tuning the ARPM threshold. **d** Plot of f1-score versus X in 1:X stratified sampling used to tune the amount of negative (non-miR) loci used in training the HRPF.
(TIF)

S8 Fig Dicer knockdown data. **a** Scatter plot for hairpins in the MCF7 (total) set, plotting log fold change of Dicer knockdown vs wildtype against the miRWoods decision value for annotated (red) and novel (blue) hairpins. The vertical line in the plot represents the decision value cut-off with all miRWoods predicted precursors to the right of it. **b** Box plot showing the log fold change of Dicer knockdown vs wildtype of annotated precursors within miRBase and novel precursors is predicted by each software for the MCF7 (total) set. **(c-d)** CDF’s for **c** MCF7 (Total) and **d** MCF7 (cytoplasmic) log fold change of Dicer knockdown vs wildtype for novel precursors.
(TIF)

S9 Fig Individual Examples of Dicer knockdown. Predicted secondary structures for **a** hsa-Novel35, **b** hsa-Novel28, **c** hsa-Novel23, **d** hsa-Nove65, **e** hsa-Novel92, and **f** hsa-Novel99. **(g-k)** RNAseq for **g** hsa-Novel35, hsa-Novel28, **h** hsa-Novel23, **i** hsa-Novel65, **j** hsa-Novel92, and **k** hsa-Novel99.
(TIF)

S10 Fig Additional Euler plots comparing miRWoods, miRDeep, and miRBase. Euler plots for **a** Human MCF7 (total), **b** Human cell lines, **c** Mouse brain, **d** Mouse embryo, **e** Mouse newborn, and **f** Mouse testes sets.
(TIF)

S11 Fig Additional Euler plots comparing miRWoods, miReap, and miRBase. Euler plots for **a**. Human MCF7 (total), **b** Human MCF7 (cytoplasmic), **c** Human cell lines, **d** Human liver, **e** Mouse brain, **f** Mouse embryo, **g** Mouse newborn, **h** Mouse testes and, **i** Mouse ovaries sets.
(TIF)

S12 Fig mir-548 Phylogenetic tree. Phylogenetic tree showing expansion of the mir-548 precursor family in human. Annotated mir-548 precursors are shown in blue and predicted novel precursors are shown in green.
(TIF)

S13 Fig Differential expression analysis of miRs not shown in main text. Expression of fca-mir-1-1 using **a** RNAseq and **b** qPCR validation of differential expression in muscle. **(c-d)** Expression of fca-mir-205 using **c** RNAseq and **d** qPCR validation of differential expression in skin.
(TIF)

S1 Table Feature correlation. A table of correlations between features used by miRWoods.
(XLSX)

S2 Table Duplex-focused spans. Percentage of cases where duplex method produced span used in final prediction.
(DOCX)

S3 Table Data summary. A summary of all of the datasets tested by miRWoods.
(XLSX)

S4 Table Mature counts. A table of raw counts, adjusted counts, reads per million (RPM), and adjusted reads per million (ARPM) for all annotated and predicted microRNA over all test sets.
(XLSX)

S5 Table Tuning experiments. Summary of data sets and results from the tuning experiments.
(XLSX)

S6 Table Dicer knockdown enrichment. P-values computed from comparing the log-fold change of dicer knockdowns compared to wild-type using a t-test, for the novel predictions of each method and miRBase.
(DOCX)

S7 Table Clusters. Clusters of annotated and predicted microRNA for human, mouse, cat, and cow genomes. miRBase was used as the annotated set for human, mouse, and cow, and the predictions from Lagana *et al*. [19] were used as the annotated set for cat. In column B under cluster_source, an annotated set was named as the source if a cluster of two or more annotations could be clustered together without miRWoods predictions. Otherwise the cluster source was called novel.
(XLSX)

S8 Table Novel families. A list of candidate novel families found after comparing predictions across all testing sets.
(XLSX)
