# Supplementary material for: miRWoods: Enhanced precursor detection and stacked random forests for the sensitive detection of microRNAs
Source: PLoS Comput Biol. 2019 Oct 9;15(10):e1007309. doi: 10.1371/journal.pcbi.1007309 (PMC6785219; doi:10.1371/journal.pcbi.1007309)
Supplement: S1 Methods — A document of providing additional detail for methods used for miRWoods. (DOCX) [file pcbi.1007309.s001.docx]

**Supplementary Methods for:**

**miRWoods: enhanced precursor detection and stacked random forests for the sensitive detection of microRNAs**

Jimmy Bell^1,^*, Maureen Larson^2^, Michelle Kutzler^3^, Massimo Bionaz^3^, Christiane V. Löhr^2^, and David Hendrix^1,4^*

^1^ School of Electrical Engineering and Computer Science, Oregon State University, Corvallis, OR, 97331, Benton Country

^2^ Departments of Clinical and Biomedical Sciences, College of Veterinary Medicine, Oregon State University, Corvallis, OR, 97351, USA

^3^ Department of Animal and Rangeland Sciences, Oregon State University, Corvallis, OR, 97331, Benton Country

^4^ Department of Biochemistry and Biophysics, Oregon State University, Corvallis, OR, 97331, Benton Country

* To whom correspondence should be addressed. Tel 1(541)737-6224. Email: david.hendrix@oregonstate.edu

Cutadapt

Adaptors were trimmed using cutadapt with the options: cutadapt -e 0.2 -m 17 -q 10 -a <adaptor>. The -e option allows a 20% error in the adaptor sequence to be cut, the -m option removes all sequences less than 17 nucleotides in length, and the -q option removes any nucleotides from the 3′ end with quality scores less than 10 prior to cutting. The -a option is used to specify an adaptor. The sequencing data are filtered further using a custom script called fastqAvgQualityFilter.pl to filter out reads with an average quality score less than 30.

Bowtie

Reads were aligned to the genome using bowtie(1) with the options: bowtie -n 1 -e 50 -l 18 -m 10 -a -S --best --strata. The -l option specifies a seed size of length 18, the -n option allows for 1 mismatch within the seed, the -e option allows for a total mismatch quality score of 50, the -m option suppresses all mappings where reads hit more than 10 places in the genome, -a sets bowtie to report all valid alignments based on the previously mentioned options, --best and --strata set bowtie to report only the best stratum of alignments, and -S sets bowtie to output the mapped reads in SAM format. After mapping, a custom script named addNHTags.pl was used to record the number of mappings for every read so that read counts could be adjusted by the number of mappings in later analysis. In some alignments the value for bowtie’s -e parameter allowed for long strings of low-quality mismatches along the 3′ end of the alignment. In order to trim the 3′ ends further a custom script called trimMirReads.pl computes the binomial probability of nucleotides being in a mismatch region and trims the read at nucleotides where the p-value is greater than 0.27.

Alignment Methods used with other Tools

For miRDeep2(2), FASTQ files were combined and reads were aligned using miRDeep2's alignment script with the options: mapper.pl <fastq> -e -i -j -k <1^st^ 6 chars of adapter> -l 18 -m -p <genome> -s <processed_reads.fa> -t <mapped_reads.arf>. The –e option specifies that a FASTQ file is used. The -i option converts rna to a dna alphabet. The -j option removes reads that have character other than a,c,g,t,u,n,A,C,G,T,U,N. The -k option sets adapter sequence to cut. The -l option sets mapper.pl to discard reads smaller than the value specified by this option. The -m option sets the script to collapse the reads. The -p option sets the genome to map to. The –s options sets the FASTA output file for processed reads. Finally, the -t option sets the output file for the reads mapped by bowtie.

For miReap (https://github.com/liqb/mireap), FASTQ files were collapsed into FASTA files and reads were aligned using the following option: bowtie -f -n 1 -e 80 -l 18 -m 10 -a -S --best --strata. In this case the -e option is set to 80 rather than 50 because bowtie sets the quality scores for character in FASTA sequences to 40. This allows for up two mismatches in the alignment and is similar to the typical number of mismatches when -e 50 is set and quality scores are present. Output from bowtie was converted into a map file that miReap used as input.

miRWoods Pipeline

miRWoods uses a stacked random forest strategy. First a random forest called the mature product random forest (MPRF) is used to filter out unlikely microRNAs. This is done to speed up the processing time required by the next step, which folds candidate microRNA with the surrounding region into hairpins and scores them. Candidate hairpins are passed to the random forest called the hairpin random forest (HPRF), which outputs the final set of predictions.

Several scripts are involved in the miRWoods pipeline. First, printReadRegions.pl is run to print a list of loci with contiguous read mappings. Next, extractProductFeatures.pl groups and scores overlapping read stacks within each locus. evaluateProductsWithRF.pl uses the MPRF to evaluate each locus and outputs a list of candidate mature microRNAs. The processReadRegions.pl script uses RNAfold(3) to fold the region surrounding the microRNA and scores them using features related to sequence, structure and the position of read stacks around the hairpin. ExtractHairpinFeatures.pl does further processing on the output of processReadRegions.pl to produce a feature vector file. evaluateHairpinsWithRF.pl runs the feature vector file through the HPRF and outputs a list of predicted microRNA precursors.

The mature product random forest (MPRF) assesses 36 features. Features related to the sequence of the mature product include median length (medianLength), Wooten Federhen complexity (WFC), GC content (GCcontent), and 16 scores assessing dinucleotide frequeny (aa,ac,ag,at,…,ta,tc,tg,tt). Features related to the distribution of reads include a measure of 5′-heterogeneity (fivePrimeHet) and 15 scores measuring the abundance of reads starting at each position between 7 nucleotides upstream to 7 nucleotides downstream of the 5′ end of the product (r7,r6,r5,…r1,s0,f1…f5,f6,f7). In order to quickly assess the viability of the fold, RNAduplex(3) is used to duplex the product with the surrounding region and measure the duplex energy. For the full defined set of features see Product Features section below.

The hairpin random forest (HPRF) predicts microRNA precursors using 71 features related to sequence, structure, and read stack positioning and read abundance around the hairpins fold. The hairpin sequence is scored by its dinucleotide frequencies (aa,ac,ag,at,…,ta,tc,tg,tt), GC content (GCcontent), and Wootton-Federhen Complexity (WFC). The scores for its major product include its unique read fraction (urf), average 5′ heterogeneity (afh), the average hit count (ahc) for its mapped reads, and the decision value returned by the MPRF (RFProductAvg.) Another feature is a count of regions with contiguous reads within 1000 bp of the candidate precursor (neighborCount.)

Several scores are used to assess hairpin structure. The product base pairing (pbp) is a score measuring the fraction of base pairings between the major product and the region spanning it on the opposite arm. The products base pairing on the opposite arm (dupPBP) is also measured along with the size of the biggest bulge or loop (dupLoopLength) and length of the sequence (dupSize.) Several more measurements cover both arms of the hairpin within the region spanning miR and miR*. These include the number of unbound nucleotides at the end of the product (maxUnboundOverhang), the largest bulge (maxBulge), the size of the largest interior loop (maxInteriorLoop), the difference in length between sides of the largest interior loop (intLoopSideDiff), and the number of additional stem-loops if the main loop is a multiloop (numOffshoots). Of course, a valid precursor would be highly unlikely if it contains a multiloop. Features relating the main loop to the rest of the hairpin included the size of the main loop (loopSize) and the distance of the miR and miR* from the main loop (mpLoopDistance and dupLoopDistance). Distances were recorded as negative in cases where the miR and miR* overlapped the main loop and zero in cases where a read stack was not present. Two features related to the energy of the structure include the minimum free energy (mfe) of the fold and duplex energy (Duplex Energy) of the major product with the surrounding hairpin. Another feature (foldDupCompare), is a measurement of the similarity of the structure returned by RNAduplex to the structure returned by RNAfold.

Several features are included that describe the abundance of reads within the hairpins. Two features measure the abundance in adjusted reads per million for reads in the sense (totalSenseRPM) and antisense (totalAntiSenseRPM) strands. In addition, miRWoods attempts to name products as miRs, moRs, loops, splits, and out products where splits are defined as read stacks overlapping a miR and loop and out products are defined as products ouside the miRs. Several features are measurements of the relative abundance for each product on both arms of the hairpin structure (rel5pOutCount, rel5pMorCount, rel5pMirCount, relLoopCount, relSplitCount, rel3pMirCount, rel3pMorCount, and rel3pOutCount).

Several features measure the variance across products. The average read positional variance (ARV) score is the average variance in start position for reads within each product. The average product variance (APV) is the variance in read counts for each distinct read of the product. These features were also weighted by the size of the products to create a weighted ARV (wARV) and weighted APV (wAPV) score. Although measures of variance were very similar all appeared to produce better accuracy when used together than when only a subset of them was used.

Several features were used to score overlaps over the entire hairpin. The sameshift score is the maximum amount that one product’s 5′ end is shifted away from an overlapping product’s 5′ end for all products in a hairpin. The bothshift score is amount of shift between two products on opposite arms of the hairpin. Features named overlap product abundance (OPA), average overlap amount (averageOverlapAmount), and total relative overlap amount (totalRelativeOverlapAmounts) scores are described by the following equations:

1. $OPA= \max_{0\leq i<j\leq n} \boldsymbol{1}_{\left[ overlap\left( i,j \right)>2 \right]}*\frac{A_{i}}{\sum_{k=0}^{n} A_{k}}$
2. $averageOverlapAmount=\sum_{i=0}^{n} \sum_{j=i+1}^{n} overlap\left( i,j \right)*\frac{A_{i}}{\sum_{k=0}^{n} A_{k}}$
3. $totalRelativeOverlapAmount=\sum_{i=0}^{n} \sum_{j=i+1}^{n} overlap\left( i,j \right)*\frac{A_{i}}{A_{j}}$

where $i,j$, and $k$ are numbers given to products in order from least abundant to most abundant, $A_{i}$ , $A_{j}$ , and $A_{k}$ is the adjusted read count of products $i, j$, and $k$, and $overlap(i,j)$is the number of nucleotides overlapping in product $i$ and product $j$. The indicator function is depicted as $\boldsymbol{1}_{[x]}$, which returns 1 if it’s argument $x$ is true, and 0 otherwise. Additional features were used to score the overlap between individual named pairs of products (miRmoR5pOverlap, miRmoR3pOverlap, miRLoop5pOverlap, miRLoop3pOverlap, loopLoopOverlap, out5pOverlap, outOut5pOverlap, outOut3pOverlap, inProdOverlap, miRSplit5pOverlap, miRSplit3pOverlap.)

The average antisense displacement (AAPD) and total antisense displacement (TAPD) features were used to measure the displacement of products overlapping one another in the sense and antisense strands. The total sense displacement score was the total displacement of the start positions of antisense products relative to the sense products they overlap. The AAPD is found by dividing the TAPD by the number of overlapping antisense products. For the full defined set of features see Hairpin Features section below.

Feature Importance with Boruta Algorithm

Boruta was used to determine the importance of features. Frequency of occurrence of reads with the same starting position as the majority of the reads in the read stack and duplex energy were the most important features in the MPRF (S1a Fig). The score from the MPRF, the ARPM in both the sense and antisense strands and the product base pairing were found to be the most important features in the HPRF (S1b Fig).

Further Feature Interpretation

The importance of features can be affected when they are highly correlated making interpretation of importance difficult. For this reason, we performed a least squares regression of the positive instances in the training set and identified correlated features with an R^2^ value greater than 0.5 (S1 Table). We trained models with correlated features removed in both the mrpf and hrpf and created a dot plot comparing them to the default version of miRWoods (see S2 Fig). A Boruta analysis was performed on the MRPF (S2a Fig) and HRPF (S2b Fig) to give an idea of what features were the most important when correlated features are used.

We plotted the change in importance for each feature after removal of the correlated features with the highest importance (S3a Fig). In some cases, features gained the importance after removal of their correlated partner after removal. In other cases, such as “dupLoopDistance” showed a substantial increase in importance despite not being correlated with other features. Removal of these redundant features did not result in a substantial reduction in performance (S3c Fig).

Effects of Abundance-Related Features on miRWoods

Several features in miRWoods are derived from read abundance, which motiviates the evaluation of whether the model can predict hairpins with low abundance. Several histograms were created comparing the number of miRs founds within human MCF-7 (total cell content) at different abundances by miRWoods, miRDeep2, and miReap (S4a-c Fig). We created a similar histograms comparing samples from mouse embryo (S4d-f Fig). In both cases, miRWoods correctly identified more microRNA loci with only one read. Taking this idea further, we created a bar chart comparing each tissue in human and mouse evaluated two out of four human samples, miRWoods consistently correctly predicted more annotated miRs with only one read compared to miRDeep2 and miReap (S4g Fig).

In order to test the performance of miRWoods without abundance-related features an additional set of models were trained removing features related to size (S4h Fig). For the MRPF, read counts at positions r7,r6,…,r1,s0,f1,…,f6,f7 were converted into fractional read counts. Features removed in the HRPF were APV, wAPV, totalSenseRPM, and totalAntiSenseRPM. The models were retrained and a scatterplot was created comparing them to the default version of miRWoods, demonstrating that while abundance-related features don’t overwhelm performance, they are important for overall prediction accuracy (S4h Fig).

Duplex Method

miRWoods considers several spans with differing genomic start and stop sites for each hairpin precursor and chooses the one with the greatest HPRF score in its final prediction. Spans are generated in two ways. The first method inputs the major product and a region spanning 75 nt on each side of the major product into RNAduplex. We refer to the span covering the major product and its duplex as the duplex-focused span. If this span partially overlaps a product, the span is elongated on both sides by the amount it would to take to overlap the other product. Following this an extra 2 nt is added to the span. The second set of spans are referred to as product-focused spans. These span between the major product and any products at least 4nt away from the major product.

A script was used to determine how often the duplex-focused span was used in the final prediction. The script generated spans using only the duplex focused method which were compared to the final miRWoods predictions (including True Positives and False Negatives.) Accuracy for each span was determined using the methods found in “Analysis of Predicted Precursors.” For many precursors the duplex-focused span was the only candidate available, so additional statistics were gathered only for predictions in which more than one candidate span was available.

The final prediction from miRWoods for annotated microRNA hairpin precursors used the duplex-focused span 88.54% of the time. The accuracy of the duplex-focused spans alone was 95.98%, and the accuracy of the spans for the final predictions from miRWoods was 96.23%. For annotations with more than one possible span, miRWoods predicted the duplex an average of 77.51% of the time for all sets. The accuracy of the spans predicted using just the duplex-focused span was 96.96%, and the accuracy of the spans for the final predictions from miRWoods was 97.39% (S2 Table).

In some cases, predictions from miRWoods were able to correct the region spanning the hairpin precursor. For instance, the annotation for hsa-mir-6860 has a precursor span that doesn’t cover an additional nearby read stack. The prediction from miRWoods covers the read stack and is along the intron boundary, showing evidence that hsa-mir-6860 is really a half-mirtron (S5a Fig).

In other cases, predictions from miRWoods showed an improvement over annotations from other software. For instance, miRWoods predicted a better hairpin span for hsa-mir-431 than miRDeep2 (S5b Fig). However, it should be noted that the miRDeep2 prediction for hsa-mir-431 had a score of 1.5 whereas the cut-off was set at 4.

The best model was therefore the result of training on human, and was evaluated on both other human samples and mouse. Remarkably, cross-species evalutions (trained on human, tested on mouse) performed better than same-species evaluations trained on human, tested on human (S6 Fig).

Data Set Overview

Small RNA sequencing data for human and mouse sets were downloaded from GEO. Data from GSE31069 were divided into 4 sets containing a wild-type and dicer knockdown version of total cell content and cytoplasmic fractions of MCF-7 cell lines. miRWoods was only run on the wild type sets, and dicer knockdown sets were analyzed to provide further evidence of dicer processing in precursors predicted in the wild type sets. GSE16579 consisted of RNAseq from several cancer cell lines and all human cell samples were combined into a single set. The three normal human liver samples from GSE21279 were combined as well. Mouse sets were all obtained from GSE20384 and were divided into brain, embryo, newborn, ovary, and testes sets. Cow samples were obtained from the lab of Dr. Massimo Bionaz and included bone marrow, dental pulp, iris, optic nerve, oral papillae, penis, retina, SLN, brain stem, and corium. Cow miRs were predicted from a combined set of all 10 tissues. Two muscle and two skin samples from cat were provided by the lab of Dr. Christiane Lohr, and miRWoods was run on the combined set. All data sets used in this study are summarized in S3 Table.

Analysis of Predicted Precursors

An analysis script was developed to determine whether the final predicted span of each hairpin precursor overlapped the annotation well enough to be considered a positive hit. To ensure fairness, predictions generated by each software were put into a common format so that the same set of functions could be used to verify them. Criteria for determining correctness of predictions are as follows:

- - For annotations with 2 products, predicted precursor span must overlap both annotated products
  - For annotations with 1 product, predicted precursor span must be within the span of the annotated precursor or half of the predicted precursor span must cover the annotation.
  - Only Predictions overlapping an annotation on the same strand (rather than the antisense strand) are deemed to be correct if they meet the above criteria.
  - If one prediction meets the above criteria, but another prediction was already given the same annotation it is counted as a false positive.

For each software pipeline, predictions meeting the above criteria were labeled as true positives and the remaining predictions were labeled as false positives. Another script was run to find all microRNAs expressed within each dataset. In this way, a consistent set of false negatives could be determined for each software by subtracting the number of correctly folded predictions from the number of expressed miRs in each dataset.

Precision, recall, and F1-score were calculated using the following equations:

(4) $Precision= \frac{True Positives}{True Positives+False Positives}$

(5) $Recall= \frac{True Positives}{True Positives+False Negatives}$

(6) $F_{1}Score=2$·$\frac{Precision\cdot Recall}{Precision+Recall}$

Summary of mature microRNA Expression

For a summary of the expression levels of all mature miRs across all samples discussed in this paper, see S4 Table.

Tuning miRWoods

Tuning the miRWoods models was done using a grid-search to train models built from GSE23090 or GSE40499. Each model was tuned on either a set of cancer cells from GSE20592 and GSE18381, or stem cells from GSE62501 and GSE65706 (S7a Fig). A final validation was done on the set that wasn’t tuned on (either the cancer cells or stem cells.) Our final model was created by training on the GSE23090 set and using the optimum tuning of decision value (S7b Fig), 1:X stratified sampling (S7c Fig), and ARPM (S7d Fig) based on the optima found for the cancer cell tuning set. A summary of the data sets and values resulting from the tuning experiment is provided in S5 Table.

Analysis of Dicer Knockdown Sets

Samples of MCF7 (total) and MCF7 (cytoplasmic fraction) with Dicer knocked down were compared with wild-type variations of the same cells. The log fold change values of in the ARPM expression in Dicer knockdown vs wildtype cells were computed for miRBase annotations and novel predictions from each software using the following equation with a pseudocount of 0.15:

(7) $Log Fold Change= {log}_{2}\left( \frac{Dicer Knockdown ARPM+pseudocount}{Wildtype ARPM+pseudocount} \right)$

A cumulative distribution function (CDF) was plotted comparing the log fold change of Dicer knockdown vs. wildtype for each software for both the MCF7 (total) and MCF7 (cytoplasmic) samples (S8a-b Fig). A scatterplot was generated for MCF7 (total) comparing the log fold change to the decision value (S8c Fig) In addition, a box plot was generated showing the median and range of the Dicer knockdown log fold change for each software tested on the MCF7 (total) set (S8d Fig). P-values were computed using a t-test comparing the log fold change in expression in Dicer-knockdowns to wildtype for the novel predictions for each method and miRBase (S6 Table).

For several novel predictions in which Dicer was knocked down the structure and RNAseq were analyzed to provide evidence for their validity. Examples included hsa-Novel35, hsa-Novel28, hsa-Novel23, hsa-Novel65, hsa-Novel92, and hsa-Novel99 (S9 Fig).

Euler Plots

Predictions from miRWoods were compared with predictions of other software using Euler diagrams. For each Euler plot, predictions intersected with the expressd microRNAs from miRBase R21 for that tissue if they met the criteria in the “Analysis of Predicted Precursors” section. Novel predictions from two programs intersected if there was any overlap at all between their predicted hairpin spans. If a program predicted two hairpins with overlapping spans which both overlap with a single case then only one of the predictions was used to increase the count at the intersection and the other prediction only increased the count for the program. Euler diagrams were plotted using the venneuler version 1.1-0 package in R (Additional Euler Plots for sets not shown in the main text can be found in S10 Fig, S11 Fig).

Phylogenetic Trees

Phylogenetic trees were generated for newly predicted and annotated versions bta-mir-2284/2285, hsa-mir-548, and fca-let7 miRs. Sequences were aligned using Clustal Omega(4) version 1.2.4 with the default parameters and the sequence type option set to DNA “-t DNA.” Next Clustal version 2.1 was used with the “-TREE” option set to create a neighbor-joining phylogenetic tree from the alignment. Circular trees for bta-mir2284/2285 and hsa-mir548 were drawn using ggtree 1.6.11 in R. The phylogenetic tree for fca-let7 family miRs was drawn using the newick package in latex. A phylogenetic tree not shown in the main text was created showing the expansion of the hsa-mir-548 family (S12 Fig).

Analysis of Clusters

For each genome known annotations and novel miRWoods predictions within 10,000 bp of each other were grouped into clusters. Clusters which could be produced by grouping only the annotated microRNA were referred to as annotated clusters. New clusters were referred to as novel cluster. For cat, the set of microRNAs predicted by Lagana *et al*. (5) were used in lieu of miRBase annotations. All identified clusters are summarized in S7 Table.

Differential expression Analysis

Differential expression was analyzed comparing muscle to skin for all mature cat miRs with an ARPM of at least 0.5 across at least 2 of the samples. The voom method was used to analyze the trend in the mean-variance relationship for the log-ARPM of the counts and limma empirical Bayes was used to build a linear model. Differential expression was determined based on the Benjamini-Hochberg adjusted p-values ($p \leq0.05$) for each miR.

Examples of differentially expressed miRs in cat included fca-mir-1-1 and fca-mir-205. RNAseq and qPCR were both used to verify that fca-mir-1-1 and fca-mir-205 were differentially expressed (S13 Fig).

PCR validation for Cow microRNA

We selected bta-Novel225-5p-miR, bta-Novel286-3p-miR, bta-Novel144-3p-miR, bta-Novel250-5p-miR, and bta-Novel244-5p-miR for qPCR validation based on decision value, structure, and positioning of read stacks.

Novel Families

Novel families were found across all sets of miRWoods predictions. In order to avoid analyzing redundant predictions, precursor spans having an overlap of 80% or more between sample sets were combined and given a single alias by which they could be identified. The sequences used for mature products came from whichever set had an instance of the precursor with greatest abundance measured in ARPM.

The combined set of novel mature microRNAs were blasted against a database containing the same novel predictions as well as all annotated microRNAs found in miRBase R21. Novel families were grouped according to the following criteria:

- major products were not homologous to known miRs
- major products were on the same side of the hairpin and shared the same seed
- Every major product in the family blasted to at least one other major product with an expect value of at least 0.05.
- Major products did not all share the exact same sequence
- Families containing only two precursors that are antisense to one another were excluded.

For a table containing all novel families of microRNA meeting these criteria see S8 Table.

**Overview of Product Features**

*(36 features total)*

**fivePrimeHet**

The five-prime-heterogeneity of the product, which is defined as the fraction of reads with 5′ start positions different from the most frequent 5′ position.

**medianLength**

The median length of reads associated with the product.

**gcContent**

the ratio of Guanine and Cytosine to all nucleotides in the product sequence

**aa, ac, ag, at, ca, cc, cg, ct, ga, gc, gg, gt, ta, tc, tg, and tt**

Frequency with which each dinucleotide appears in the product sequence.

**r7,r6,r5,r4,r3,r2,r1,s0,f1,f2,f3,f4,f5,f6, and f7**

The abundance of reads with start positions that are between 7 nucleotides upstream to 7 nucleotides downstream of the 5′ end of the product

**WFC**

Wooten-Federhen Complexity of the product sequence defined for a sequence with $n_{A}\ldots n_{U}$ for the counts for the number of occurrences of A, C, G, and U respectively as:

$$C_{WF}=\frac{1}{N}{log}_{D}(\frac{N!}{n_{A}!n_{C}!n_{G}!n_{U}!})$$

Where $N$ is the total number of nucleotides in the sequence, and $D=4$ is the size of the alphabet.

**Duplex Energy**

The minimum free energy for the product duplexed with the surrounding sequence (returned by RNAduplex.)

**Hairpin Features**

*(71 features total)*

**mfe**

the minimum free energy of the folded hairpin (returned by RNAfold)

**tapd**

sum of the displacement of the 5′ end of the sense products to the 3′ end of the antisense products (antisense products only add to the TAPD if their abundance is at least 1/2000 of the hairpin abundance and 1/20 of the abundance of the corresponding sense product.)

**aapd**

The average antisense displacement score. Found by dividing the tapd by the number of antisense products.

**urf**

The unique read fraction. Found by dividing the number of unique reads in the hairpin by the number of adjusted reads (adjusted reads are normalized by the number of mappings to the genome.)

**ahc**

The average max product hit count. The average number of hits to the genome of all distinct reads with start positions 10 nucleotides up or downstream of the miR product.

**afh**

The average max product 5′ heterogeneity. The frequency of reads that do not line up with the 5′ end of the miR.

**pbp**

Product base pairing. The frequency of paired bases on the miR.

**sameShift**

sameShift is the maximum amount that one product’s 5′ end is shifted away from an overlapping product’s 5′ end for all products in a hairpin. (This is computed only if the amount of overlap between the products is greater than 2, the ratio of the two products is greater than 1/20, and the read counts of both products are greater than 1% of reads in the hairpin.)

**bothShift**

The maximum amount that two products on opposite arms of the hairpin are shifted from each other. (This is computed only if the amount of overlap between the products is greater than 2, the ratio of the two products is greater than 1/20, and the read counts of both products are greater than 1% of reads in the hairpin.)

**OPA**

Overlap Product Abundance. The frequency of the most abundant product that overlaps another product by more than 2.

**aa, ac, ag, at, ca, cc, cg, ct, ga, gc, gg, gt, ta, tc, tg, and tt**

Dinucleotide content scores defined as the frequency of occurrence.

**Duplex Energy**

The value for minimum free energy returned by RNAduplex.

**GCcontent**

the ratio of Guanine and Cytosine to all nucleotides in the sequence

**foldDupCmp**

This score compares the structure produced by RNAfold with the structures produced by RNAduplex. The degree to which they are similar is divided by the total length of the RNAduplex structures. For example:

RNAduplex structure: (.(.((((((((&).)))))..))).) Total len = 26

(.(.(((((((( ).)))))..))).)

+++((((.(.((((((((.((..........))))).))))).)))).))+++++++++

Total matched = 25

foldDupCmp = 25/26 = 0.962

**dupPBP**

duplex Product base Pairing. The product base pairing of the region duplexed by the miR. The pbp of the duplex may be different from the pbp of the miR due to extra loops and bulges. In these cases, the dupPBP feature may aid in correctly classifying non-miRs.

**dupLoopLength**

The size of the biggest interior bulge or loop in the duplex across from the major miR product.

**APV**

Average Product Variance. The average variance of the read counts for distinct reads within a product.

**wAPV**

Weighted Average Product Variance. This is the same as APV but weighted by the size of each product.

**ARV**

Average Read Positional Variance. The average variance in the start positions of the reads for each product.

**wARV**

Weighted Average Read Positional Variance. This is the same as ARV but weighted by the size of each product.

**mpLoopDistance**

The distance of the miR from the loop. Can be negative if the miR overlaps the loop.

**dupLoopDistance**

The distance of the miR* from the loop. Can be negative if the miR* overlaps the loop.

**loopSize**

The size of the loop

**averageOverlapAmount**

For each overlapping pair of products, the read count of the less abundant product is multiplied by the amount of overlap, then divided by the total read count of the hairpin. The results of these computations are summed to give the score for the averageOverlapAmount.

**totalRelativeOverlapAmount**

For each overlapping pair of products, the read count of the less abundant product is multiplied by the amount of overlap, then divided by the read count of the more abundant product. The results of these computations are summed to give the score for the totalRelativeOverlapAmount.

**innerLoopGapCount**

Counts the number of times more three or more unbound nucleotides are next to each other in the Loop region.

**totalSenseRPM**

The total adjusted reads per million (ARPM) in the sense strand

**totalAntisenseRPM**

The total adjusted reads per million (ARPM) in the antisense strand

**totalOverlap**

The sum of the amounts of overlap between each pair of overlapping reads.

**maxBulge**

The longest set of unbound nucleotides in the region of the hairpin spanning the miR and miR* products.

**maxInteriorLoop**

The biggest interior loop in the region of the hairpin spanning the miR and miR* products.

**intLoopSideDiff**

The difference between the length of the interior loop between the miR and miR* products

**maxUnboundOverhang**

The largest amount of unbound nucleotides on either side of the miR

**numOffshoots**

The number of additional hairpins formed on or across from the miR or miR*. If there are any it is not likely to be a real mir precursor.

**dupSize**

The size of the region duplexed by the miR product. If it is really small (due to overlapping a loop) or really large (due to there being several hairpins or atypical folding) it is unlikely to be a miR.

**neighborCount**

The number of read regions that are within 1000 nucleotides of the precursor. If the precursor is within an intron, only the positions within the intron are checked and the neighborCount is adjusted to an amount comparable to what would be expected within 1000 nucleotides. This is similar to nonMiRNeighborCount in miRTRAP(6) but includes all read regions.

**RFProductAvg**

The Decision value returned by the random forest used in the product phase.

**rel5pOutCount, rel5pMorCount, rel5pMirCount, relLoopCount, relSplitCount, rel3pMirCount, rel3pMorCount, and rel3pOutCount**

the fraction of the product relative to the total for the hairpin

**miRmoR5pOverlap, miRmoR3pOverlap, miRLoop5pOverlap, miRLoop3pOverlap, loopLoopOverlap, out5pOverlap, outOut5pOverlap, outOut3pOverlap, inProdOverlap, miRSplit5pOverlap, and miRSplit3pOverlap**

Overlaps for individual products within the loop. These are negative if products are a distance from one another.

References

1. Langmead B, Trapnell C, Pop M, Salzberg SL. Ultrafast and memory-efficient alignment of short DNA sequences to the human genome. Genome biology. 2009;10(3):R25.

2. Friedländer MR, Chen W, Adamidi C, Maaskola J, Einspanier R, Knespel S, et al. Discovering microRNAs from deep sequencing data using miRDeep. Nature biotechnology. 2008;26(4):407.

3. Lorenz R, Bernhart SH, Zu Siederdissen CH, Tafer H, Flamm C, Stadler PF, et al. ViennaRNA Package 2.0. Algorithms for Molecular Biology. 2011;6(1):26.

4. Sievers F, Higgins DG. Clustal Omega, accurate alignment of very large numbers of sequences. Multiple sequence alignment methods: Springer; 2014. p. 105-16.

5. Laganà A, Dirksen WP, Supsavhad W, Yilmaz AS, Ozer HG, Feller JD, et al. Discovery and characterization of the feline miRNAome. Scientific Reports. 2017;7(1):9263.

6. Hendrix D, Levine M, Shi W. miRTRAP, a computational method for the systematic identification of miRNAs from high throughput sequencing data. Genome biology. 2010;11(4):R39.
