# Supplementary figures and images for: miRWoods: Enhanced precursor detection and stacked random forests for the sensitive detection of microRNAs
Source: PLoS Comput Biol. 2019 Oct 9;15(10):e1007309. doi: 10.1371/journal.pcbi.1007309 (PMC6785219; doi:10.1371/journal.pcbi.1007309)

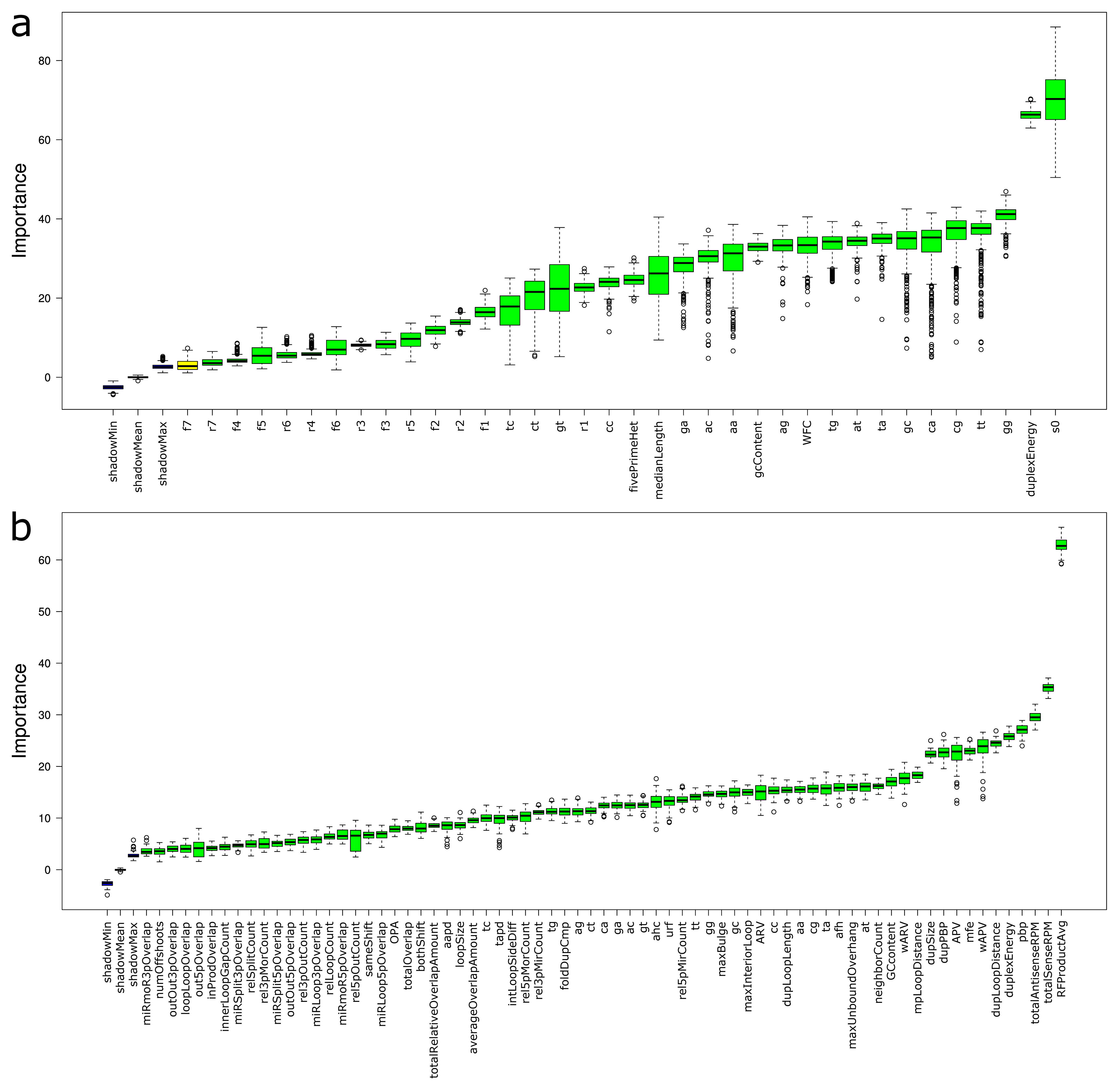

Supplement: S1 Fig — a The importance of each feature based on the Boruta analysis for the mature product random forest (MPRF) b The importance of each feature based on the Boruta analysis for the Hairpin Random Forest (HPRF). (TIF) [file pcbi.1007309.s002.tif]

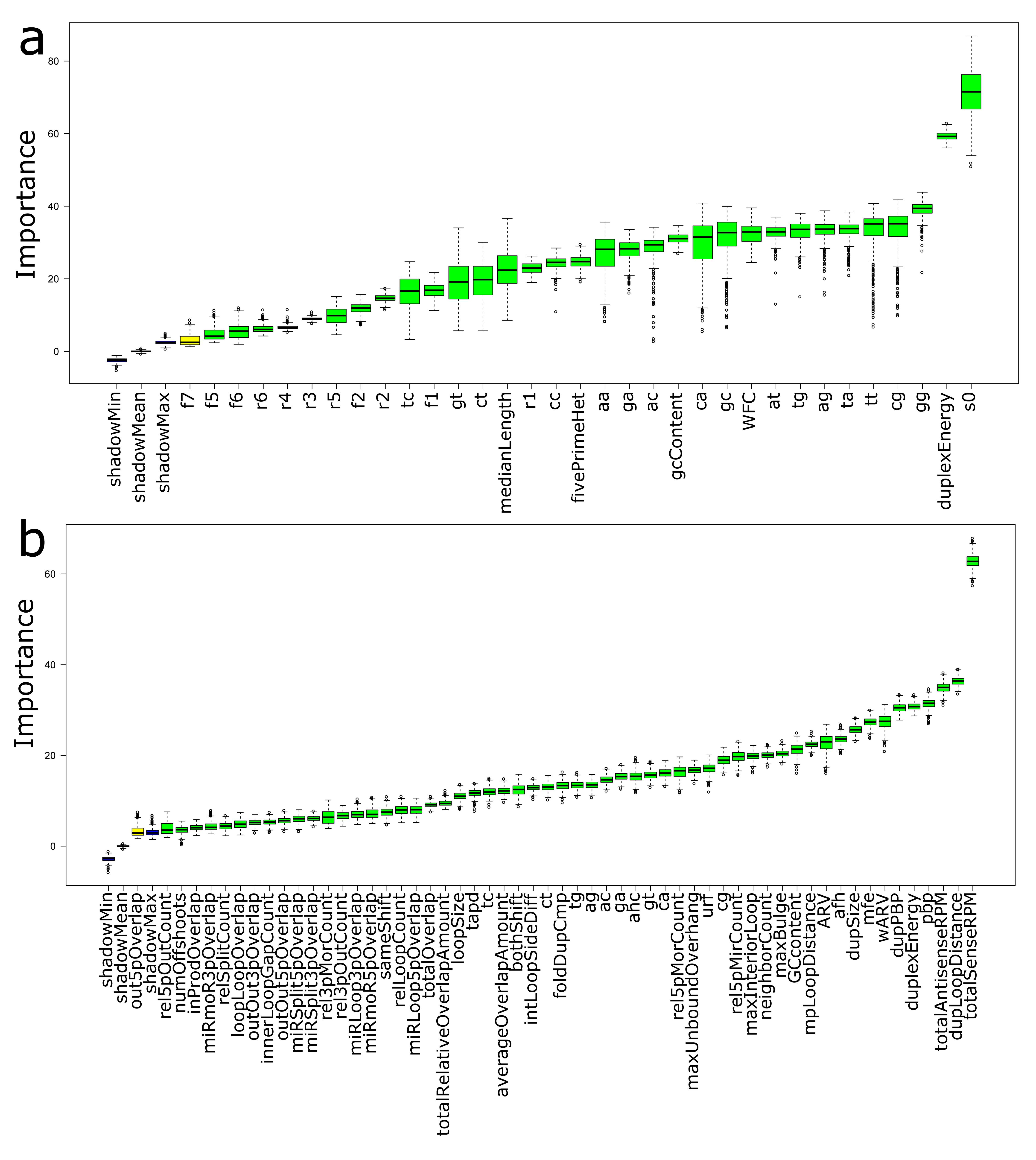

Supplement: S2 Fig — a Boruta analysis of feature importance for MRPF with correlated features removed. b Boruta analysis for HRPF with correlated features and the MRPF decision value removed. (TIF) [file pcbi.1007309.s003.tif]

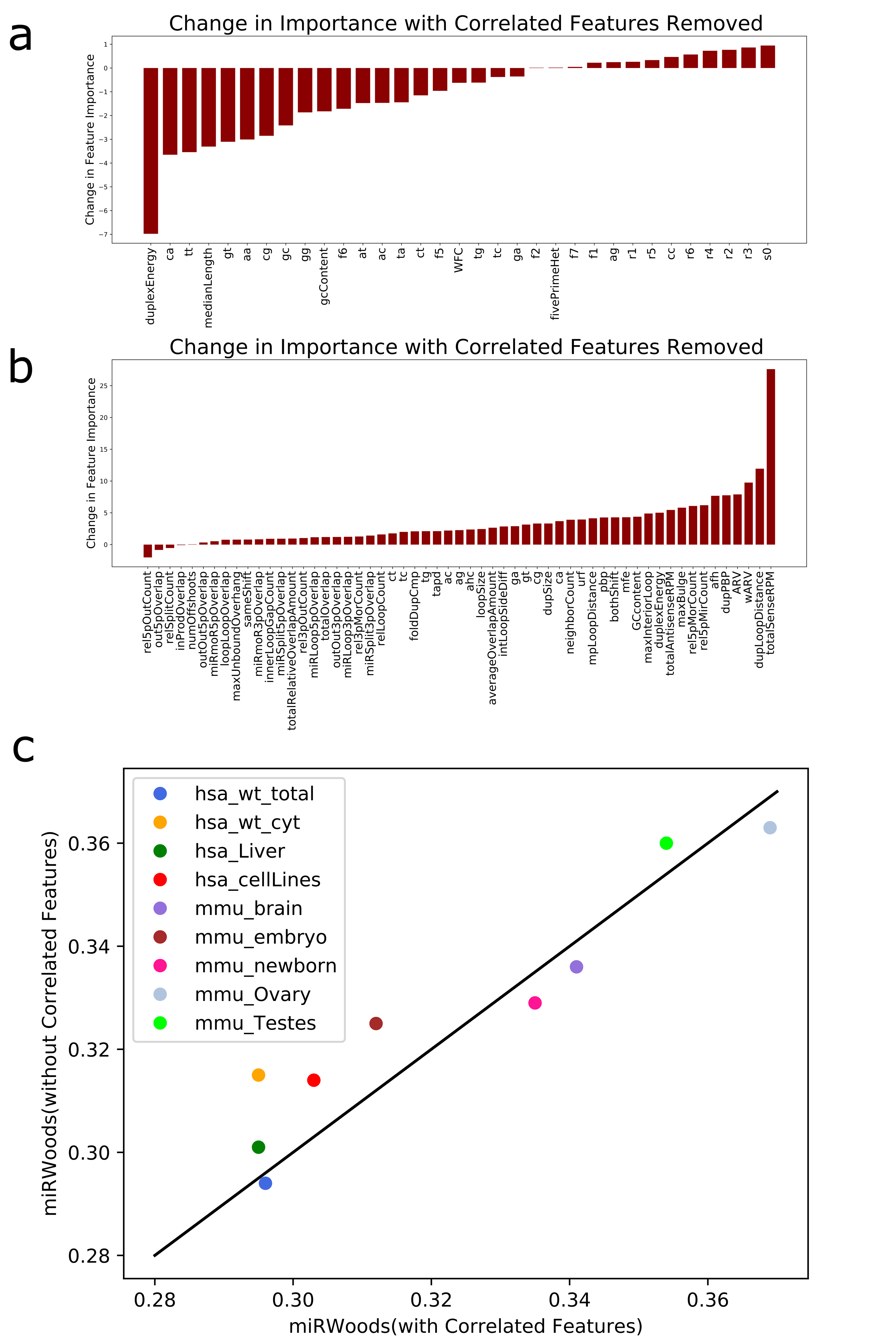

Supplement: S3 Fig — a Change in feature importance for MRPF with correlated features removed. b Change in feature importance for HRPF with correlated features removed.c the F1-score for miRWoods with correlated features and MRPF decision value removed compared to the full feature set. (TIF) [file pcbi.1007309.s004.tif]

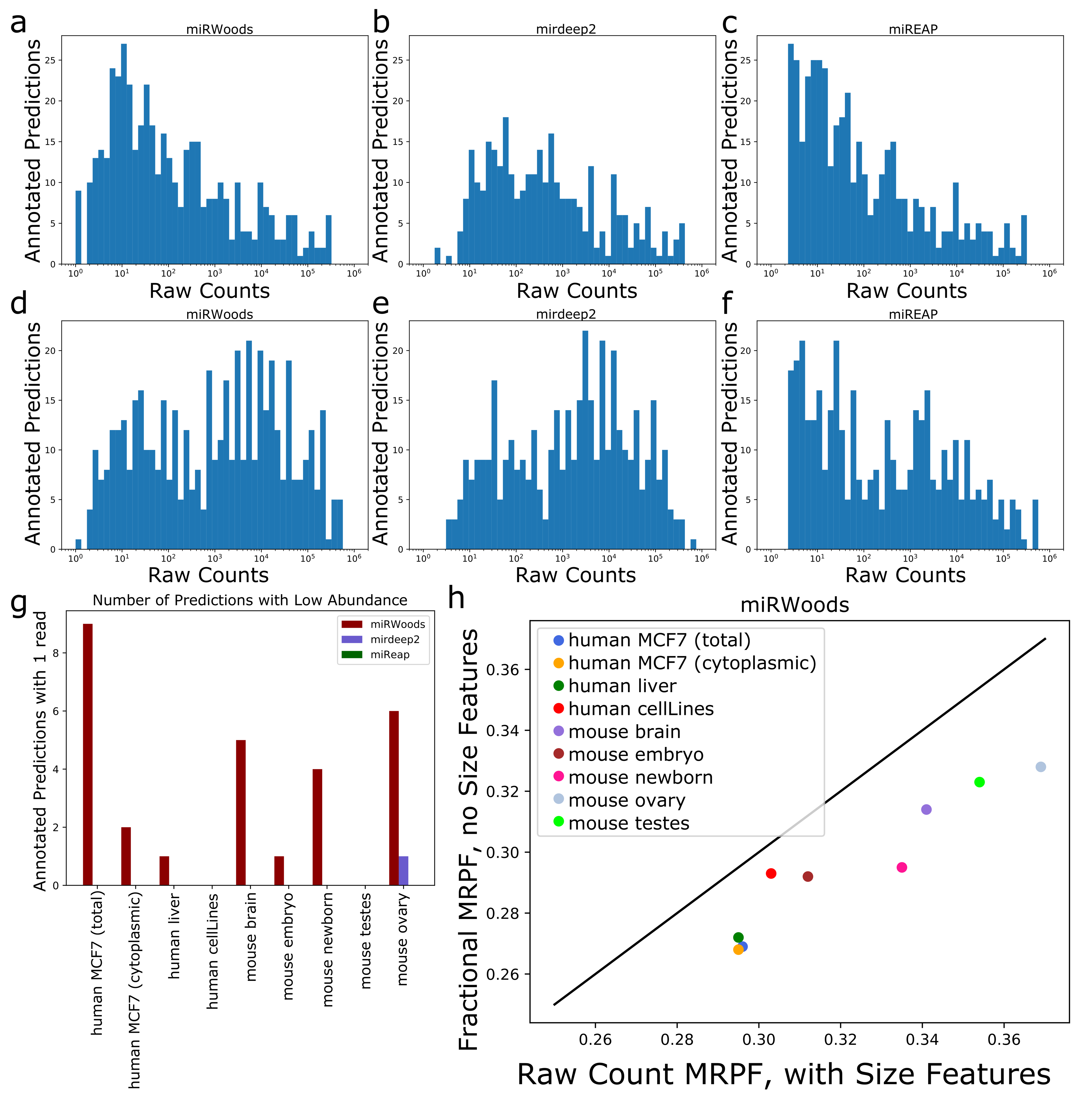

Supplement: S4 Fig — a Distribution of read abundance for correct miRWoods predictions on MCF7 total cell content. b distribution of read abundance for correct miRDeep2 predictions on MCF7 total cell content. c distribution of read abundance for correct miReap predictions on MCF7 total cell content. d Distribution of read abundance for correct miRWoods predictions on mouse embryos. e distribution of read abundance for correct miRDeep2 predictions on mouse embryos. f distribution of read abundance for correct miReap predictions on mouse embryos. g bar plot of correct predictions with only one read for all samples in human and mouse. h F1-score of predictions with size-related features compared to without. (TIF) [file pcbi.1007309.s005.tif]

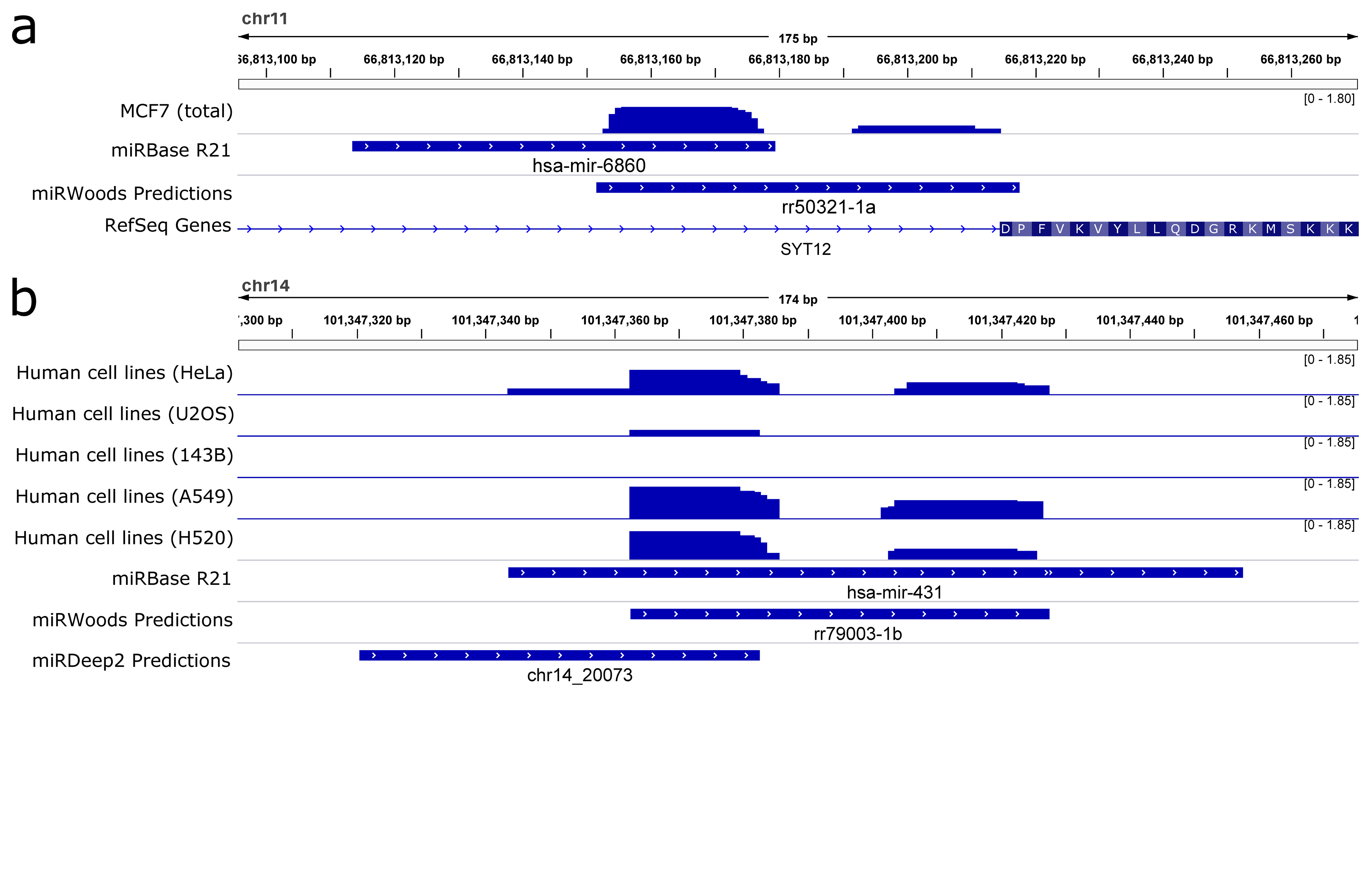

Supplement: S5 Fig — a RNAseq for hsa-miR-6860 shows miRWoods prediction covering an additional read stack next to the splice junction, which indicates that hsa-miR-6860 may be a half-mirtron. b RNAseq for hsa-mir-431 showing predicted folds for miRWoods and miRDeep. (TIF) [file pcbi.1007309.s006.tif]

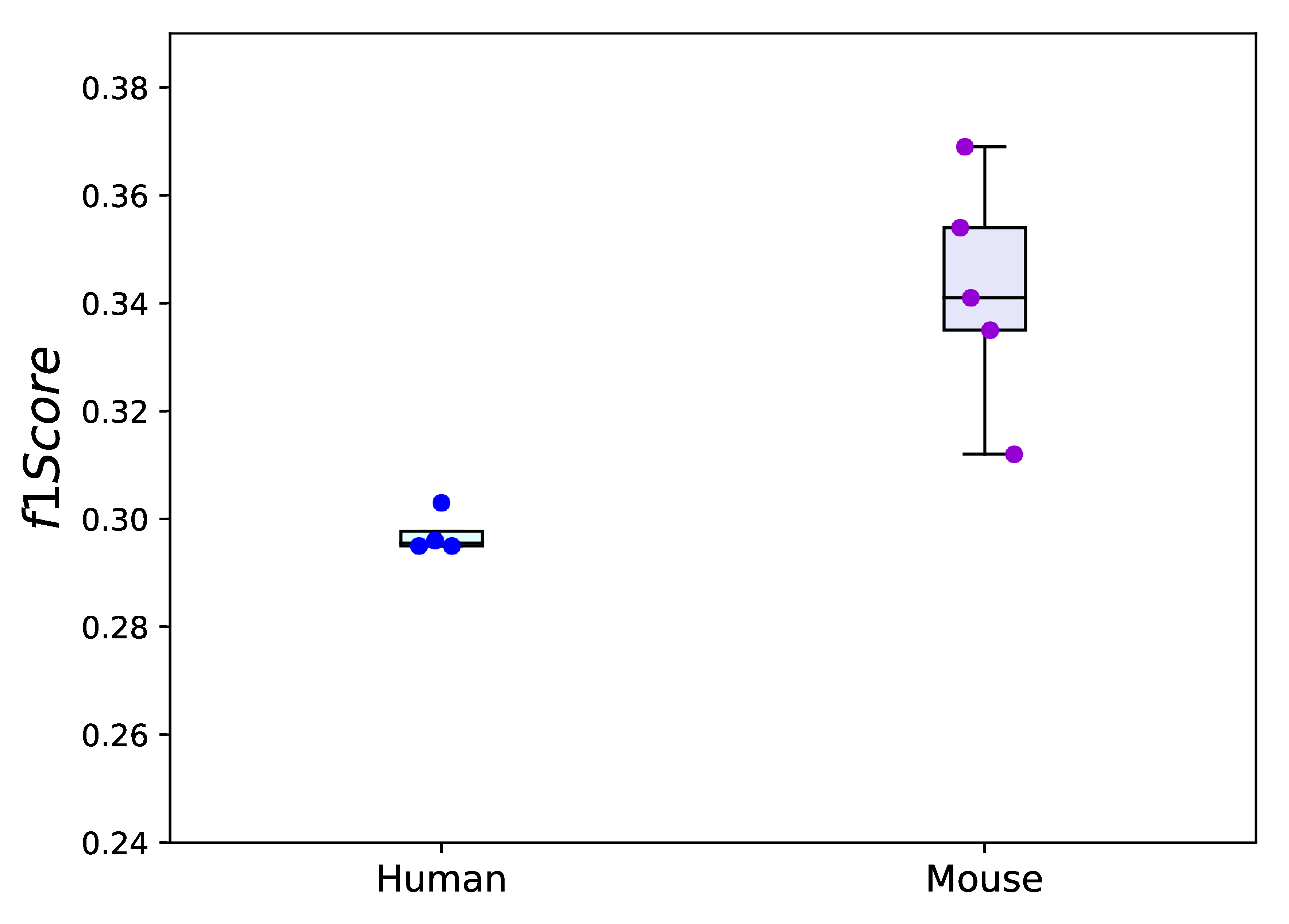

Supplement: S6 Fig — Comparison between cross-species F1-score and same-species F1-score. All of miRWoods evaluations were tested on a single model trained and tuned on human datasets. The best performance is observed on mouse samples. (TIF) [file pcbi.1007309.s007.tif]

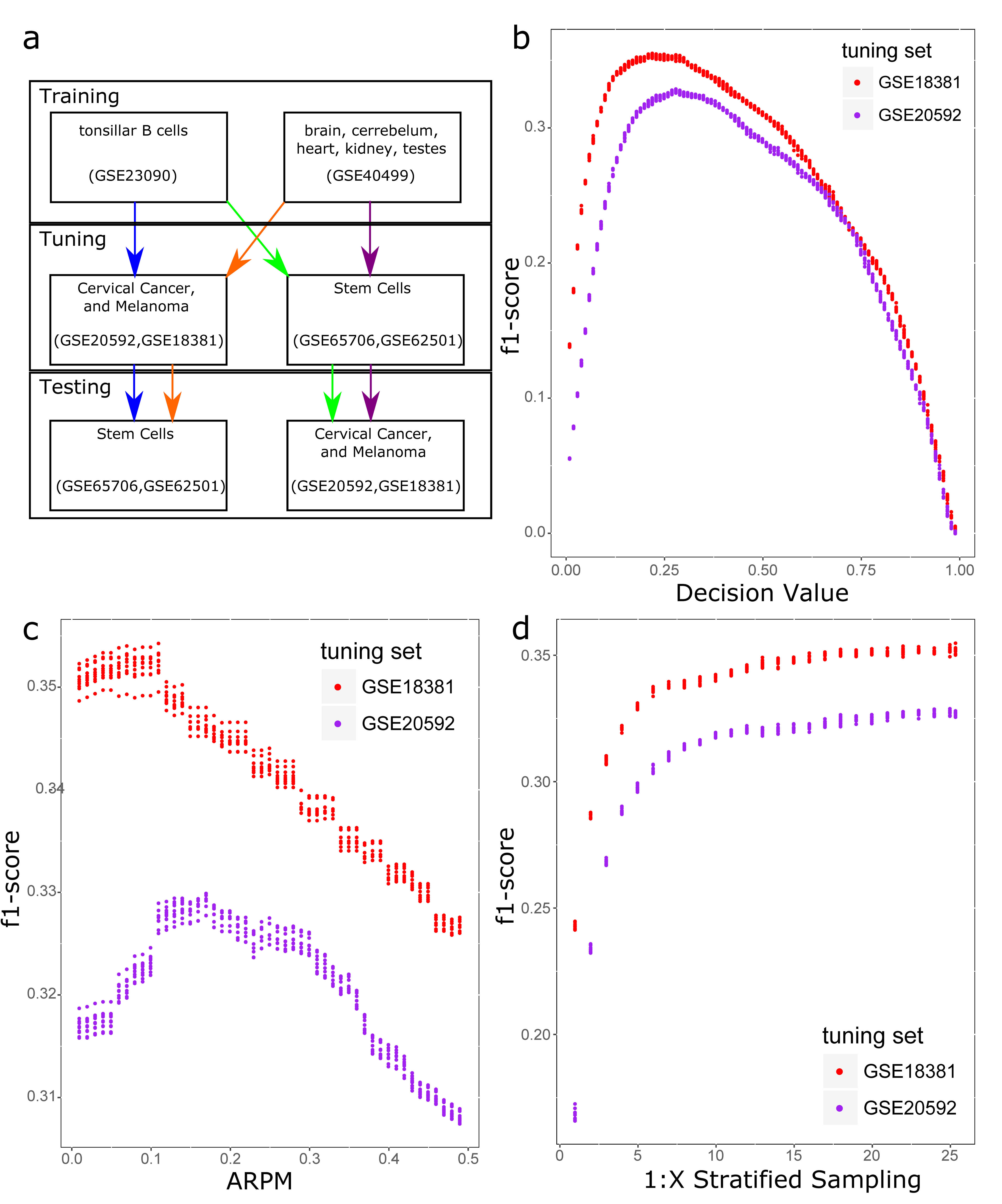

Supplement: S7 Fig — a Analysis pipelines and corresponding data sets used for training, tuning, and testing correspond to the paths of the arrows. b Plot of F1-score versus decision value threshold used in tuning the decision value threshold. c Plot of F1-score versus ARPM threshold used in tuning the ARPM threshold. d Plot of f1-score versus X in 1:X stratified sampling used to tune the amount of negative (non-miR) loci used in training the HRPF. (TIF) [file pcbi.1007309.s008.tif]

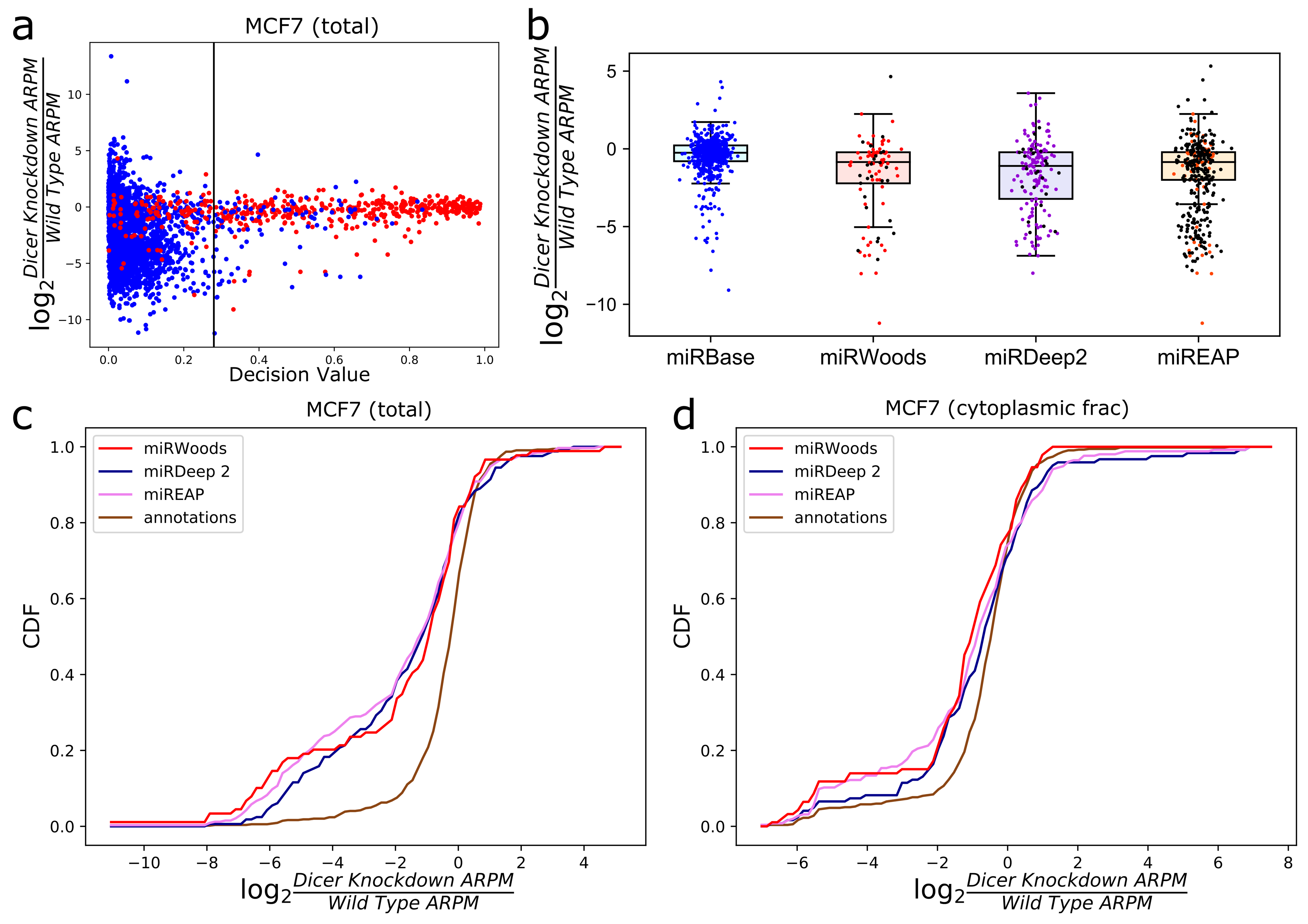

Supplement: S8 Fig — a Scatter plot for hairpins in the MCF7 (total) set, plotting log fold change of Dicer knockdown vs wildtype against the miRWoods decision value for annotated (red) and novel (blue) hairpins. The vertical line in the plot represents the decision value cut-off with all miRWoods predicted precursors to the right of it. b Box plot showing the log fold change of Dicer knockdown vs wildtype of annotated precursors within miRBase and novel precursors is predicted by each software for the MCF7 (total) set. (c-d) CDF’s for c MCF7 (Total) and d MCF7 (cytoplasmic) log fold change of Dicer knockdown vs wildtype for novel precursors. (TIF) [file pcbi.1007309.s009.tif]

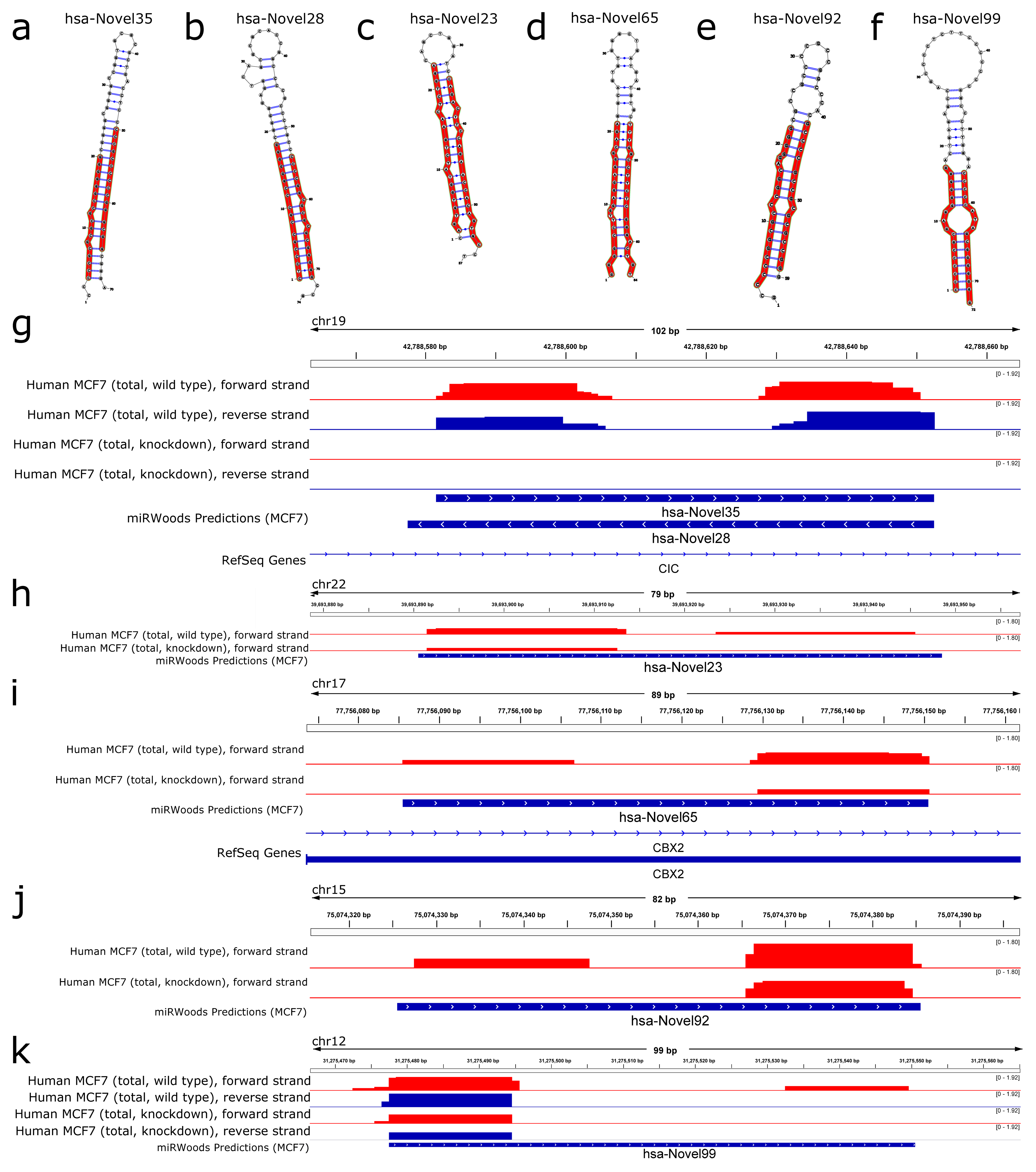

Supplement: S9 Fig — Predicted secondary structures for a hsa-Novel35, b hsa-Novel28, c hsa-Novel23, d hsa-Nove65, e hsa-Novel92, and f hsa-Novel99. (g-k) RNAseq for g hsa-Novel35, hsa-Novel28, h hsa-Novel23, i hsa-Novel65, j hsa-Novel92, and k hsa-Novel99. (TIF) [file pcbi.1007309.s010.tif]

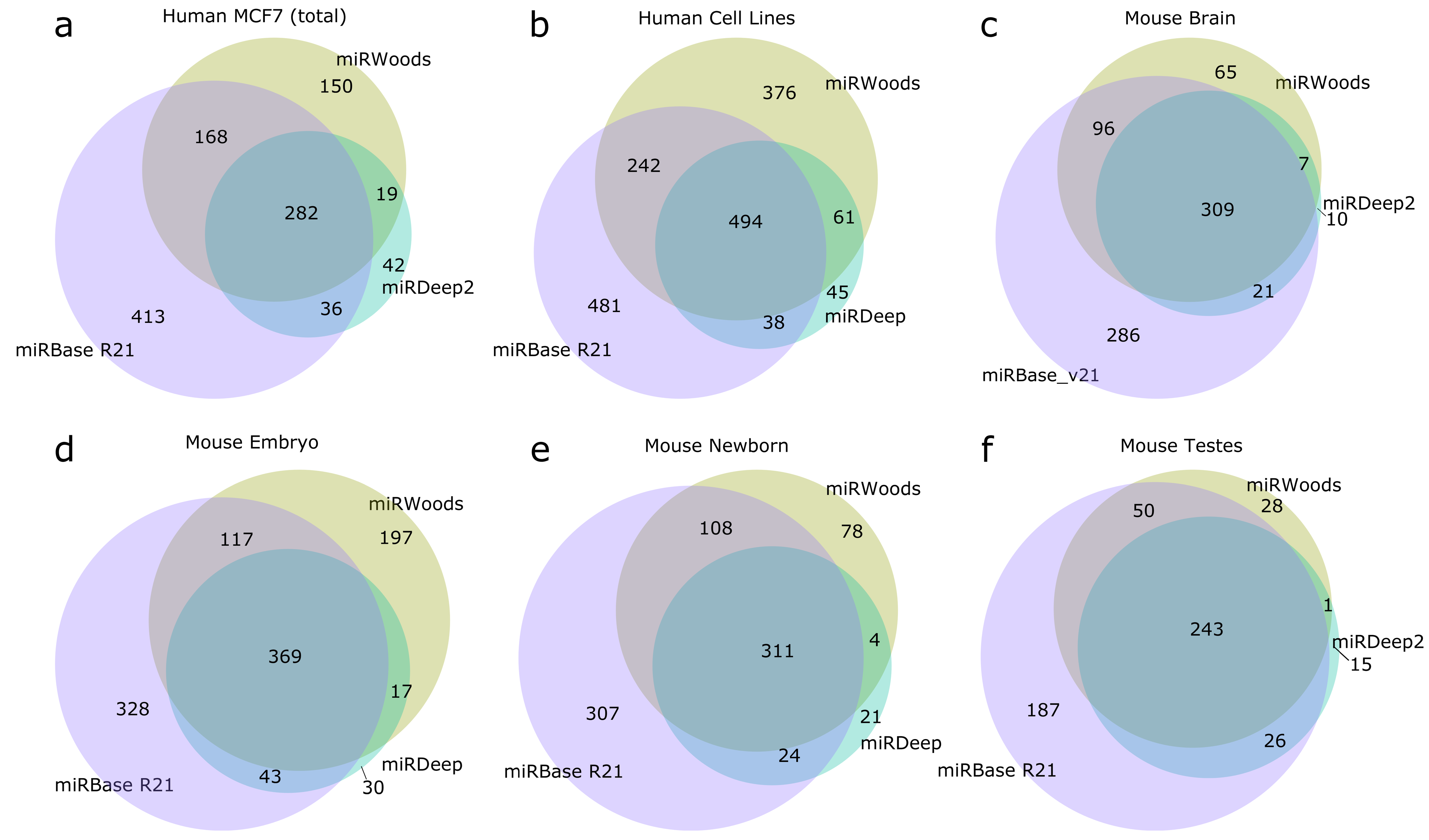

Supplement: S10 Fig — Euler plots for a Human MCF7 (total), b Human cell lines, c Mouse brain, d Mouse embryo, e Mouse newborn, and f Mouse testes sets. (TIF) [file pcbi.1007309.s011.tif]

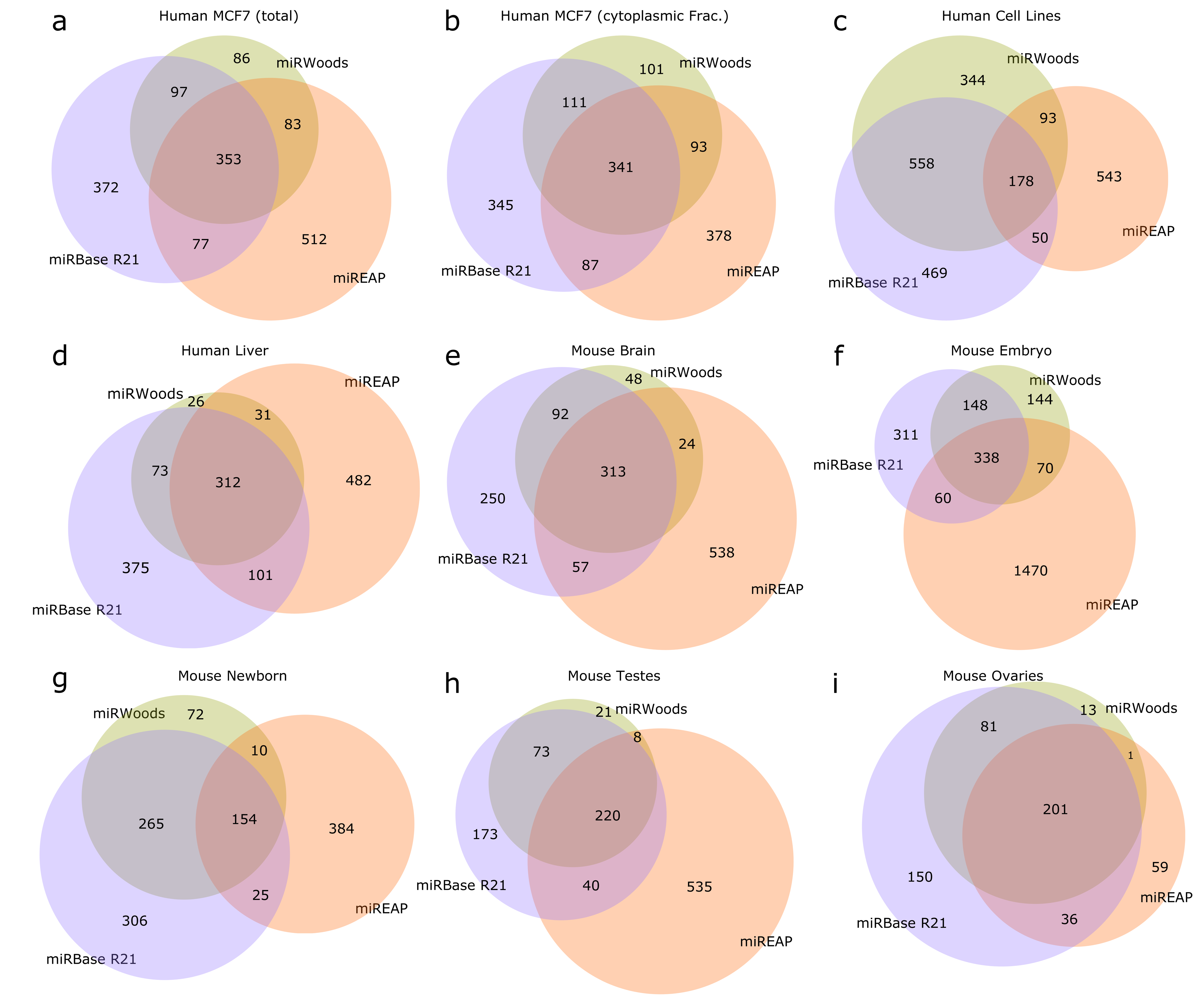

Supplement: S11 Fig — Euler plots for a. Human MCF7 (total), b Human MCF7 (cytoplasmic), c Human cell lines, d Human liver, e Mouse brain, f Mouse embryo, g Mouse newborn, h Mouse testes and, i Mouse ovaries sets. (TIF) [file pcbi.1007309.s012.tif]

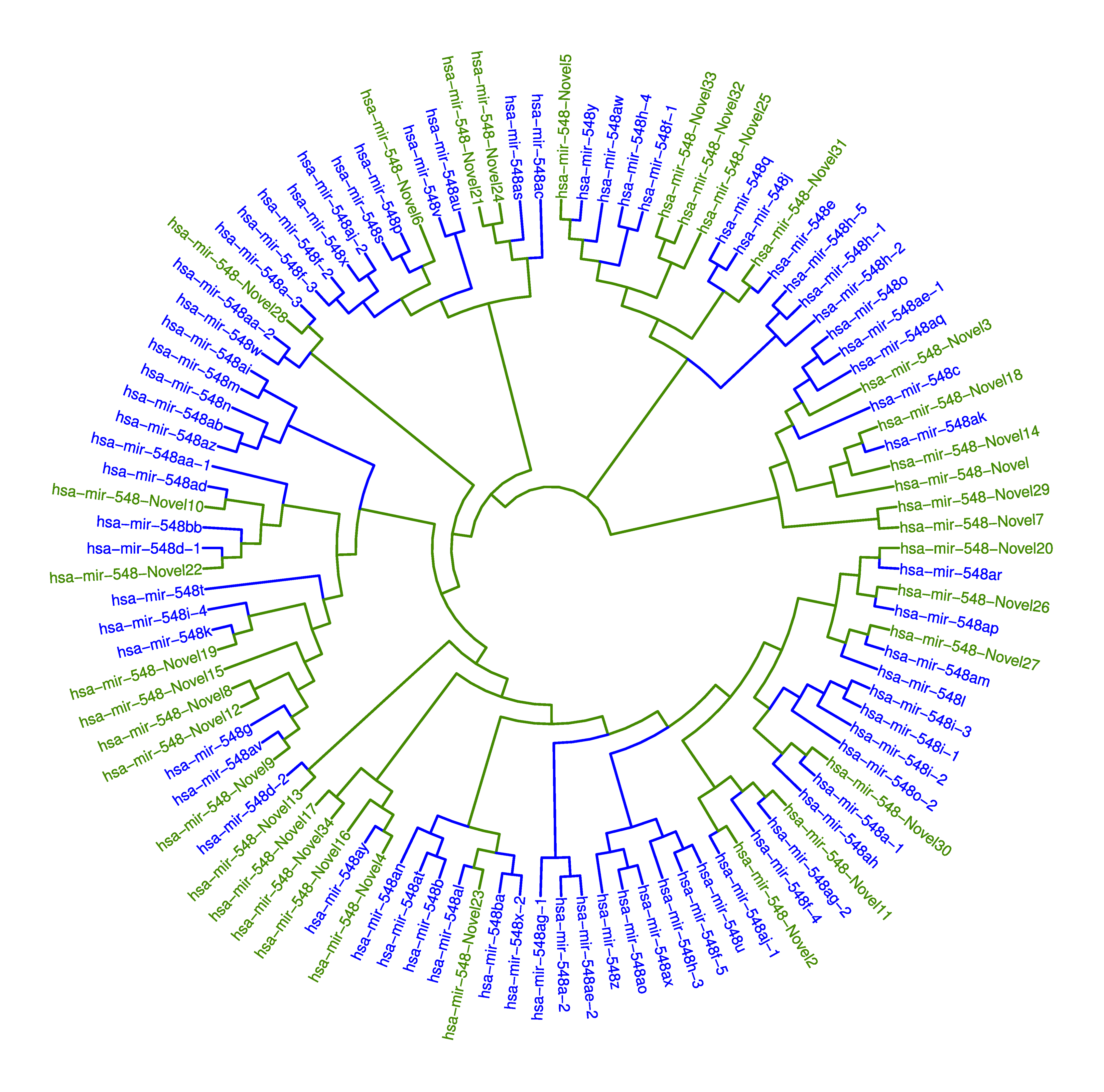

Supplement: S12 Fig — Phylogenetic tree showing expansion of the mir-548 precursor family in human. Annotated mir-548 precursors are shown in blue and predicted novel precursors are shown in green. (TIF) [file pcbi.1007309.s013.tif]

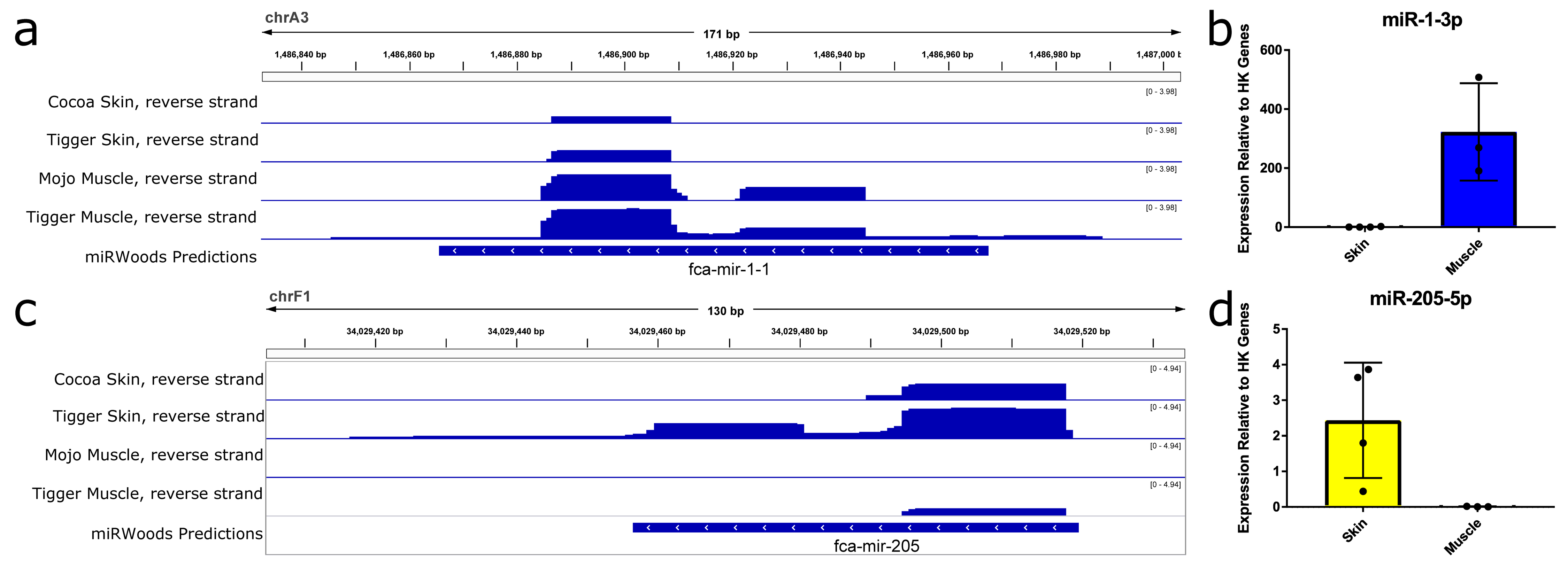

Supplement: S13 Fig — Expression of fca-mir-1-1 using a RNAseq and b qPCR validation of differential expression in muscle. (c-d) Expression of fca-mir-205 using c RNAseq and d qPCR validation of differential expression in skin. (TIF) [file pcbi.1007309.s014.tif]
