# Supplementary material for: miRWoods: Enhanced precursor detection and stacked random forests for the sensitive detection of microRNAs
Source: PLoS Comput Biol. 2019 Oct 9;15(10):e1007309. doi: 10.1371/journal.pcbi.1007309 (PMC6785219; doi:10.1371/journal.pcbi.1007309)
Supplement: S2 Table — Percentage of cases where duplex method produced span used in final prediction. (DOCX) [file pcbi.1007309.s016.docx]

Supplementary Table S2. Percentage of cases where duplex method produced span used in final prediction.

|  | All Annotated Precursors | | | Annotated Precursors with candidate spans > 1 | | |
| --- | --- | --- | --- | --- | --- | --- |
| Sample | Duplex-Focused Span Matches Predicted (%) | Duplex-Focused Span Correct (%) | Predicted Span Correct (%) | Duplex-Focused Span Matches Predicted (%) | Duplex-Focused Span Correct (%) | Predicted Span Correct (%) |
| MCF7 (total) | 90.19 | 96.56 | 96.82 | 77.49 | 97.66 | 98.25 |
| MCF7 (cytoplasm) | 88.55 | 95.97 | 96.10 | 75.41 | 97.57 | 97.84 |
| cell lines | 85.99 | 95.25 | 96.18 | 78.90 | 95.91 | 97.31 |
| Liver | 89.43 | 96.12 | 95.86 | 78.24 | 96.69 | 96.14 |
