# Supplementary material for: miRWoods: Enhanced precursor detection and stacked random forests for the sensitive detection of microRNAs
Source: PLoS Comput Biol. 2019 Oct 9;15(10):e1007309. doi: 10.1371/journal.pcbi.1007309 (PMC6785219; doi:10.1371/journal.pcbi.1007309)
Supplement: S6 Table — P-values computed from comparing the log-fold change of dicer knockdowns compared to wild-type using a t-test, for the novel predictions of each method and miRBase. (DOCX) [file pcbi.1007309.s020.docx]

Supplementary Table S6. P-values computed from comparing the log-fold change of dicer knockdowns compared to wild-type using a t-test, for the novel predictions of each method and miRBase.

| **MCF-7 Cytoplasmic Fraction** | | |  |  |
| --- | --- | --- | --- | --- |
|  | miRWoods | miRDeep2 | miReap | miRBase |
| miRWoods | - | 0.05086187 | 0.29649206 | 0.00012122 |
| mirdeep | 0.05086187 | - | 0.26416239 | 0.42224388 |
| miReap | 0.29649206 | 0.26416239 | - | 0.00296758 |
| miRBase | 0.00012122 | 0.42224388 | 0.00296758 | - |
| **MCF-7 Total Cell Content** | |  |  |  |
|  | miRWoods | miRDeep2 | miReap | miRBase |
| miRWoods | - | 0.58468605 | 0.7934435 | 1.3489E-14 |
| mirdeep | 0.58468605 | - | 0.27722909 | 6.9076E-18 |
| miReap | 0.7934435 | 0.27722909 | - | 1.9469E-28 |
| miRBase | 1.3489E-14 | 6.9076E-18 | 1.9469E-28 | - |
